# Screening for interaction effects in gene expression data

**DOI:** 10.1371/journal.pone.0173847

**Published:** 2017-03-16

**Authors:** Peter J. Castaldi, Michael H. Cho, Liming Liang, Edwin K. Silverman, Craig P. Hersh, Kenneth Rice, Hugues Aschard

**Affiliations:** 1 Channing Division of Network Medicine, Brigham and Women's Hospital and Harvard Medical School, Boston, Massachusetts, United States of America; 2 Division of General Internal Medicine and Primary Care, Brigham and Women's Hospital, Boston, Massachusetts, United States of America; 3 Pulmonary and Critical Care Division, Brigham and Women's Hospital and Harvard Medical School, Boston, Massachusetts, United States of America; 4 Department of Epidemiology, Harvard T.H. Chan School of Public Health, Boston, Massachusetts, United States of America; 5 Department of Biostatistics, University of Washington, Seattle, Washington, United States of America; 6 Centre de Bioinformatique, Biostatistique et Biologie Intégrative (C3BI), Institut Pasteur, Paris, France; University of California Irvine, UNITED STATES

## Abstract

Expression quantitative trait (eQTL) studies are a powerful tool for identifying genetic variants that affect levels of messenger RNA. Since gene expression is controlled by a complex network of gene-regulating factors, one way to identify these factors is to search for interaction effects between genetic variants and mRNA levels of transcription factors (TFs) and their respective target genes. However, identification of interaction effects in gene expression data pose a variety of methodological challenges, and it has become clear that such analyses should be conducted and interpreted with caution. Investigating the validity and interpretability of several interaction tests when screening for eQTL SNPs whose effect on the target gene expression is modified by the expression level of a transcription factor, we characterized two important methodological issues. First, we stress the scale-dependency of interaction effects and highlight that commonly applied transformation of gene expression data can induce or remove interactions, making interpretation of results more challenging. We then demonstrate that, in the setting of moderate to strong interaction effects on the order of what may be reasonably expected for eQTL studies, standard interaction screening can be biased due to heteroscedasticity induced by true interactions. Using simulation and real data analysis, we outline a set of reasonable minimum conditions and sample size requirements for reliable detection of variant-by-environment and variant-by-TF interactions using the heteroscedasticity consistent covariance-based approach.

## Introduction

Gene-gene and gene-environment interaction effects on common human traits and diseases have been difficult to identify [1]. Part of the challenge is the small effect size of genetic variants on macro-phenotypes (e.g. disease status or anthropometric traits). Assuming that interactions have effect sizes of the same magnitude as marginal genetic effects, the sample size needed to detect them can be up to an order of magnitude larger [2]. In order to circumvent this issue, researchers have performed screening for interaction effects on intermediate phenotypes (e.g., gene expression, proteomic, metabolomic) that presumably are directly affected by genetic variation in a causal pathway from variant to disease phenotype [3–6]. Indeed, reported marginal effects of single nucleotide polymorphisms (SNP) on gene expression are often substantially higher than those reported in genome-wide association studies (GWAS) of common traits and diseases. It is reasonable to assume that the interaction effects will also be larger and therefore easier to detect.

In this study we analyzed blood gene expression and genotype data from 121 subjects in the ECLIPSE Study [7, 8] to test for interaction effects between cis-eQTL SNPs (i.e. SNPs within 250kb of any autosomal gene) and the expression levels of transcription factors (TFs), since one of the known mechanisms for expression quantitative trait loci (eQTLs) is disruption of TF-binding motifs [9]. However, after careful evaluation of empirical performance of standard methods, we found that Type I error rates can be severely inflated. In particular, we show through simulations that genome-wide interaction screening in the setting of moderate to large main and interaction effects poses two major challenges. The first challenge relates to data pre-processing. Heavy pre-processing is commonly applied to gene expression data to account for variability across samples, libraries, or experimental conditions [10–12]. Choices made at this stage can impact the results of interaction screening, and while some approaches likely address specific technical artifacts more effectively, no pre-processing strategy is known to be universally best [13, 14]. Pre-processing often also includes variable normalization to obtain approximately-Gaussian data, which can help the small-sample performance of testing approaches (see e.g. [3, 4]). However, interaction effects are scale-dependent [15–17] and non-linear transformation of the data can have a major impact on the interpretation of interaction tests. The second challenge relates to statistical issues that arise in the presence of moderate to strong interaction effects. We and others showed in previous work that interactions can influence the distribution of a quantitative trait conditional on the interacting predictors [18, 19]. For small interaction effects, as expected for most human traits and diseases, the impact on the outcome distribution is expected to be minimal. However, moderate to strong interaction effects can induce substantial heterogeneity of variance by genotypic class, which can in turn lead to inconsistent covariance matrix estimation. Non-constant variance can induce uncontrolled Type I error rates and decreased power. This implies that the presence of a strong interaction between two predictors (e.g., a SNP and a TF) can potentially invalidate screening for interaction effect between the interacting SNP and other risk factors.

Using simulation we investigate these issues by quantifying the performance of five analytical strategies to detect interaction: standard linear regression, two heteroscedasticity-consistent covariance estimates, dichotomizing the predictors, and a saturated model. More specifically, we assessed the robustness of these approaches when heteroscedasticity has been induced through interaction effects while varying sample size, minor allele frequency and the magnitude of the main and interaction effects simulated. We identify minimal conditions necessary for valid tests of interaction in eQTL studies. Finally, for illustration purposes, we also present the results from the TF by SNP interaction screening in ECLIPSE. This real data analysis confirms the findings of our simulations, highlighting that standard approaches might have severely inflated type I error rate. Moreover, we observed that the list of significant associations changed dramatically across approaches, especially when comparing analyses of transformed versus untransformed gene expression data, stressing that the two sets of analyses capture different patterns in the data.

## Results

### Impact of non-linear transformation of expression data

**Fig 1:**
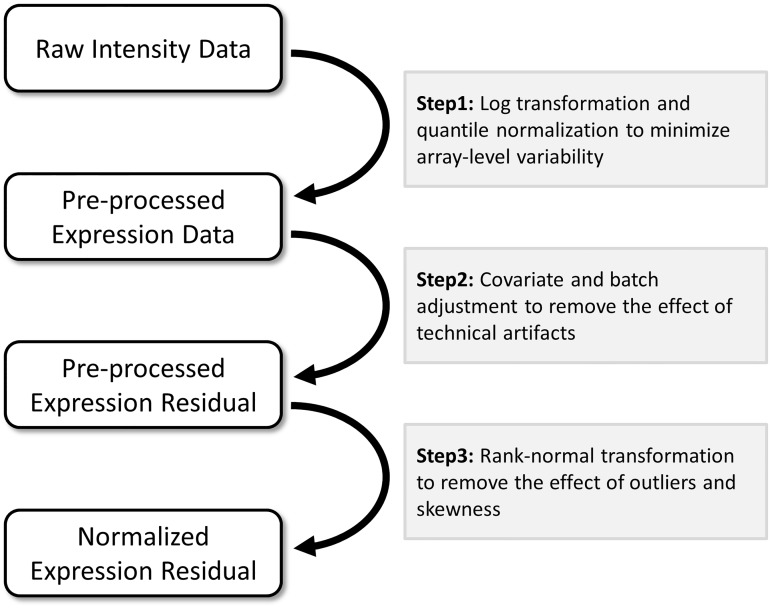
Gene expression data pre-processing pipeline. Standard pre-processing methods applied to gene expression data prior to expression quantitative trait locus analysis. Note that alternative strategies are also used. For example step 2 is sometimes skipped and confounding factors (e.g. batch) are included in the model tested as covariates. Others have also applied step 3 before step 2.

Gene expression data are heavily pre-processed (Fig 1). We assume here the first two stages of pre-processing result in the removal of most sources of technical variability. We examined the impact of non-linear transformations (*step 3* in Fig 1) and rank-based inverse normal transformations (*rkt*) in particular, as the latter approach is commonly applied in eQTL studies. Applying *rkt* on expression data can have several advantages. First, if the data show deviation from normality (e.g., has an exponential-like distribution or skewness), *rkt* can increase the statistical power to detect the linear marginal effect of a predictor. In brief, non-linear transformation can achieve a normal and homoscedastic distribution of points around the regression line, potentially allowing for a larger amount of the outcome variance to be captured, thus increasing power [20, 21]. However, this is not an absolute rule and it should be noted that *rkt* can also decrease power in certain scenarios [21]. Overall, non-linear transformations that preserve ranks are unlikely to induce false signals for marginal effects, and they are a valid alternative to non-linear regression when the distribution of the error is not normal [22]. Indeed, previous studies used ranks instead of raw data to identify upregulated genes [23, 24]. Another advantage of *rkt* is that it addresses outlying values while preserving sample size. Expression data often has noise-induced outliers (i.e., a few individuals with high expression values) because of experimental artifacts or stochastic properties of the biological system, which can lead to an increased rate of type I and type II errors [25]. The naive correction based on removing outliers can substantially reduce sample size. Rank-based normalization allows for those subjects to be retained.

**Fig 2:**
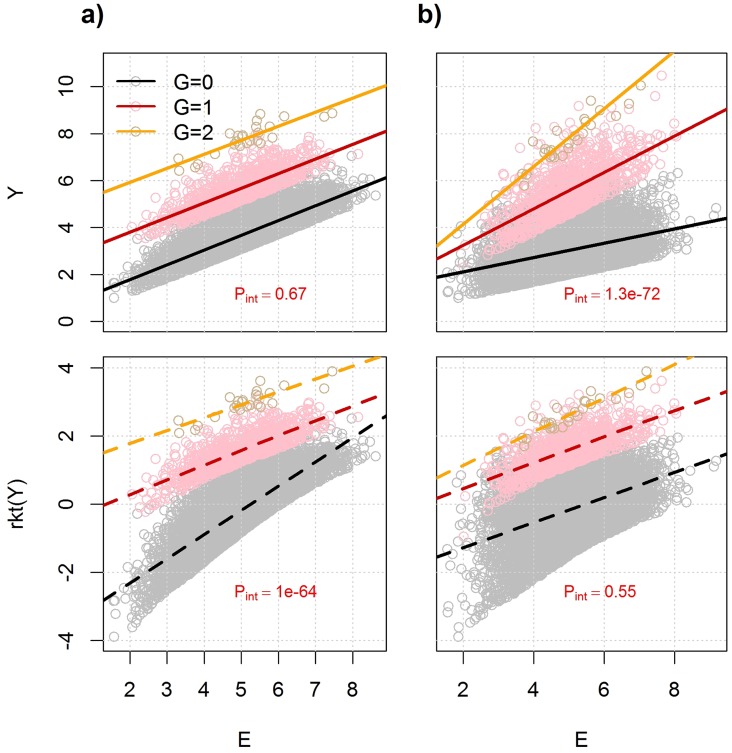
Effect of non-linear transformation on interaction effects. We defined an outcome *Y* as a function of a single nucleotide polymorphism *G* with a minor allele frequency of 0.1, an exposure *E* normally distributed with mean 5 and variance 1, and a right-skewed normal distributed residual term *ε*. In the framework of this analysis, TF mRNA level is considered as an exposure *E*. We generated two datasets of 10,000 individuals for the two scenarios. In a) *G* and *E* have only main effects and each explain 20% of the variance of *Y*. In b) *G* and *E* main effects each explain 10% of the outcome variance, but also have an interaction effect explaining 20% of the variance of *Y*. Upper panels show *Y* as a function of *E* by genotypic class and trend slope from a standard linear regression. Lower panels show the same data plotted after a rank-normal transformation (*rkt*) of *Y*. Interaction effect (observed as differences in slope by genotypic class) appears or disappears depending on the transformation applied to *Y*. *P*-values for interaction are indicated in red.

However, in contrast to screening for marginal effects, *rkt* might have a stronger impact on Type I and Type II error rates when testing for interaction [21, 26]. More generally, non-linear transformations such as *rkt* can dramatically impact the interpretation of interaction effects. In particular, while differences in significance before and after transformations are expected to be relatively minimal when interaction effects are small (e.g. *r*^2^<1%, as in genome-wide association studies of common traits and diseases), this is not necessarily the case when the variance explained by predictors is large. Consider for example the generative model defined in Eq (1) (see Material and methods) where the expression of gene *Y* depends on a SNP *G*, an exposure *E* interacting with *G* (mRNA levels of a transcription factor in our case), and the residual *ε* follows a skewed-normal distribution with mean 0 and variance 1 (in order to reflect deviation from normality as observed for some gene expression data). Note that for simplicity, we assume the covariate *Z* has no effect (*γ*_*z*_ = 0) on *Y*. In the absence of interaction effect (*γ*_*GE*_ = 0), if *E* and *G* explain a relatively large amount of the variance of *Y* (e.g., *r*^2^≥30%, see **Fig A in** S1 File, doing a rank-based inverse normal transformation of *Y* (or other type of non-linear transformation) can induce a statistical interaction between *G* and *E*. For illustration, we plotted in Fig 2 two representative scenarios of transformation inducing or removing interaction. One of the examples is where the generative model does not include interaction, while the rank-normal transform data does; and the second example illustrates the opposite case where the generative model does include an interaction between *G* and *E*, but the rank-normal transformed data does not. Note that we considered a non-normal residual as it can be an argument for applying a rank-based inverse normal transformation, but similar issues might arise when the residuals are normally distributed. **Figs B-C in** S1 File further illustrate how rank-based transformation can induce interaction when none is present on the original scale. While the scale-dependency of interaction effects has been demonstrated previously [15–17], this issue is infrequently addressed in gene expression interaction analyses. Commonly applied non-linear data transformations should be accounted for in the analysis and interpretation of interaction effects in gene expression data.

### The paradox of interaction effects

The second major challenge we found is that while weak interaction effects between two risk factors are unlikely to have a noticeable impact on the conditional distribution of the outcome, this is not the case for moderate to strong interactions. For an interacting SNP, this will be expressed through heterogeneity of variance of the outcome residual by genotypic class, a property that has been proposed as a target for interaction screening (using heterogeneity of the outcome’s variance as a proxy for the residual’s variance) [6, 18, 19]. Heteroscedasticity can cause the usual standard error estimates of ordinary least square coefficients to be inconsistent, thereby invalidating tests for interaction between that SNP and other risk factors (excluding the true interacting factor which would be under the alternative) [27]. To illustrate this point we conducted a simulation study using the model previously described (Eq (1), see Material and methods), but assuming the true interacting factor, *E*, unknown, and drawing *ε*, the residual from a normal distribution with variance scaled so that the variance of *Y* is one for all scenarios simulated. For simplicity we also assumed that *E* and *Z* follow normal distributions with mean 0 and variance 1. We tested for interaction between *G* and the non-interacting factor *Z* in a series of replicates simulated in the presence or absence of main effects for *G*, *E* and *Z*, as defined in Eq (2) (see Material and methods).

**Fig 3:**
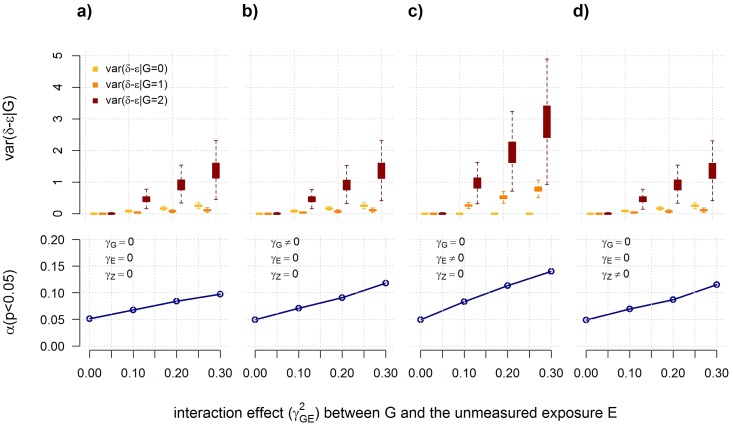
When a true interaction can bias interaction screening. A quantitative outcome *Y* is defined as a linear function of a SNP *G*, an unmeasured exposure *E*, a measured exposure *Z*, and an interaction between *G* by *E*, with effect *γ*_*G*_, *γ*_*E*_, *γ*_*Z*_, and *γ*_*GE*_, respectively (as defined in Eq 1). All predictors were standardized to have mean 0 and variance 1. In the framework of this analysis, TF mRNA level is considered as an exposure *E*. We vary *γ*_*GE*_ so that the interaction term explains between 0 and 30% of the variance of *Y*. For simplicity we assume that, when relevant, the main effect of either *G*, *E*, or *Z* explains the same amount of variance as the interaction effect and set *ε* so that the variance of *Y* equals 1. Using this model we simulated series of 10,000 replicates, each including 400 individuals and tested for interaction between *G* and *Z* using a model not including the unmeasured exposure *E* (as defined in Eq 2), in the absence of main effect of the predictors (*γ*_*G*_ = *γ*_*E*_ = *γ*_*Z*_ = 0), panel *a*) or when including a main effect of *G* (*γ*_*G*_ ≠ 0, panel *b*), a main effect of *E* (*γ*_*E*_ ≠ 0), panel *b*), or a main effect of *G* (*γ*_*Z*_ ≠ 0, panel *d*). Upper panels show the increase in the residual variance of the outcome *δ* minus *ε* (so that models are comparable) stratified by genotypic class while increasing the interaction effect *γ*_*GE*_. Lower panels show the type I error rate *α* at a *p*-value threshold of 0.05 for the interaction tests between *G* and *Z* derived for each series of 10,000 replicates.

Fig 3 illustrates how moderate to strong interaction effects can induce variance heterogeneity of the outcome residual (*δ*) by genotypic class, resulting in inflation of the type I error rate of the interaction tests between *G* and *Z*. This is in agreement with previous work, also showing type I error inflation when misspecifying the main genetic effect [28–30]. Variance heterogeneity mostly depends on the strength of the interaction effect and the main effect of the (unmeasured) exposure, although increasing main effects of the tested interacting factor (here *G* and *Z*) can also worsen the type I error inflation (Fig 3 and **Text A in** S1 File). Assuming the magnitude of the interaction effects are similar to the marginal genetic effects observed in cis-eQTL screening (e.g., *r*^2^>30%, see **Fig A in** S1 File), this analysis demonstrates that genetic variants found to have significant interactions with multiple factors should be interpreted with caution, as this may indicate that the tested SNP is involved in a strong interaction with *some* factors, but not necessarily with the factors tested. The key point here is that in a simple model, assuming covariates included in the model are not confounding factors of the predictors tested, heteroscedasticity caused by a strong interaction between the tested variant and an unmeasured factor (or at least a factor not included in the model) can induce spurious interaction effects with other non-interacting factors. Note that heteroscedasticity generated by interaction would only be a limited concern for the test of the marginal genetic effect. Indeed *G* being involved in an interaction means it is related to the outcome tested, and while power and effect estimation might be impacted, a marginal effect association signal with *G* would likely represent a true signal.

### Correcting for inflation

We then extended our simulations to assess the validity of five approaches in the presence of a strong interaction between the SNP tested and an unmeasured factor. As before, we assumed that the gene expression data have been corrected for technical artifacts but not rank transformed (*step 1* and *2* in Fig 1). We considered first standard linear regression without further correction (Eq 2). We then considered the heteroscedasticity consistent (HC) covariance-based approach. A number of alternatives have been proposed to deal with this issue [31], and while some might perform better than others, a complete comparison of these methods is beyond the scope of this study. Therefore we focused on the two most established approaches, the sandwich covariance matrix estimator (HC0) proposed by White [27], and the jackknife HC covariance (HC3), which has been suggested as the most efficient approach to deal with heteroscedasticity in small sample size [32]. Both methods are commonly used in genetic association studies [28, 33, 34]. As proposed for GWAS, we also considered two other methods that address model misspecification [30], namely dichotomizing the exposure *Z*, and using a saturated model that includes non-linear main effects of the predictors. For the latter approach we simply included in the model a main effect for *Z*^2^ and for each genotypic class (*G* = 1 and *G* = 2).

**Fig 4:**
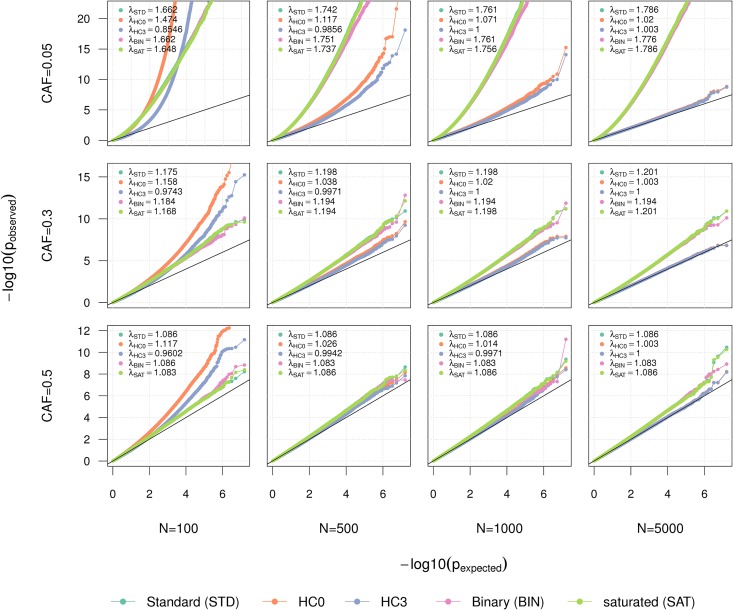
Robustness comparison. QQplots over series of 8 million replicates where an outcome *Y* is simulated as a function of a genetic variant *G*, an unmeasured exposure *E*, an interaction between *G* and *E*, and in 50% of the replicates a measured exposure *Z*. In the framework of this analysis, *Z* and *E* are considered as measured and unmeasured TF mRNA level, respectively. The validity of five tests is evaluated by comparing the observed -log_10_ (p-value) against the expected -log_10_ (p-value) when testing for the null interaction between a *G* and *Z*. The tests include a standard linear regression using main and interaction terms only (STD), heteroscedasticity consistent-based tests using effect estimates from STD (HC0 and HC3), linear regression using binary-transformed *Z* (BIN), and a saturated model including a main effect of *Z*^2^ and each genotype coded as dummy variable (SAT). We considered coded allele frequency (CAF) of 0.05 (first row), 0.3 (middle row) and 0.5 (bottom row), and sample size *N* of 100, 500, 1,000 and 5,000. We randomly draw *E*, *Z*, and *ε*, the residual of *Y* from either a normal or a right-skewed normal distribution. For each scenario we derived the genomic inflation factor *λ*_*GC*_.

We compared the robustness of the five approaches across a series of one million simulated replicates for the null model of no interaction between *G* and *Z*. We considered 96 different scenarios, increasing sample size from 100 to 5,000 (reflecting sample size in recent analyses [3, 4, 6]), increasing the coded allele frequency (CAF, analogous to minor allele frequency in this case) from 0.05 to 0.5, assuming normal or a skewed normal distribution for the three continuous variables of the experiment (*E*, *Z* and *ε*), and assuming alternatively presence or absence of a main effect for *Z*. The magnitude of the *G* × *E* interaction effects and the main effects of the predictors (*E*, *G*, and *Z* when relevant) were generated at random for each replicate. As shown in the QQplots from **Fig D-K in** S1 File, sample size and CAF had the strongest impact on the results, while we observed minor differences when varying the other parameters. Fig 4 presents the average performance of the five tests across the two former parameters (the sample size and CAF). This simulation shows that tests for interaction in small sample sizes (<100) are subject to strong type I error rate inflation for all studied methods. This inflation decreases with increasing sample size, however it can remain substantial for low frequency variants. Moreover, the inflation is non-linear, meaning that genomic control (GC) correction [35] cannot ensure the validity of the test. Overall, HC3 had the best performance, displaying an inflation factor *λ* close to 1 and only minor or no inflation of low p-values when using a sample size of 1000 or greater and common SNPs (CAF ≥ 0.3).

### Interaction effect screening in ECLIPSE

To illustrate these effects in real data, we conducted a two-step screening approach to identify interaction effects between cis-eQTL SNPs and candidate TFs in a genome-wide expression dataset from 121 subjects in the ECLIPSE Study [36]. Interaction triplets (SNP, TF and target gene) were defined based on an *a priori* list of TFs and significant SNP-gene pairs from the ECLIPSE cis eQTL analysis. We followed the pre-processing procedure of Fig 1, except for part 3 (i.e., no rank-based inverse normal transformation). Step one consisted of testing all SNPs in the vicinity of a gene for *cis*-effect on the expression of that gene assuming an additive genetic effect and using a score test as implemented in the GGTools software [37], and to select the most significant cis-eQTL as candidate SNPs for interaction effect testing. Among the 18,834,685 gene-SNP pairs tested, we identified 132,074 SNPs with a *q*-value for association less than or equal to 0.01. From these results we selected the most significant SNP for each Affymetrix probe set, resulting in a total of 2,982 *cis*-eQTL SNPs associated with 3,334 *target* probe sets. As observed in other studies, the variance of the residualized expression phenotypes explained by *cis*-eQTL SNPs can be much larger than those observed for quantitative phenotype in human GWAS [38]. **Fig A in** S1 File presents the distribution of *r*^2^ obtained from marginal genetic model for the 3,334 pairs. The average equals 0.45 with a maximum *r*^2^ of 0.87. In comparison, under a null model with the same total number of tests and using the same *p*-value threshold, we would expect to observe a distribution of effects to have a mean *r*^2^ of 0.14 and a maximum of approximately 0.25.

In step two, all *cis*-eQTL SNPs selected at step one were tested for interaction with the expression level of candidate TFs obtained from the publication by Vazquerizas et al [39]. To reduce the multiple testing burden, candidate TFs were tested for interaction with a given gene only if their marginal association with the *target* probe set was nominally significant (*p*<0.05). Also, to avoid confounding by a *cis*-effect of a SNP on two genes in close physical proximity, all TFs within 10Mb of a candidate *cis*-eQTL SNP were not tested for interaction with that SNP and its target gene. Among 1494 TFs, 1292 TFs represented by 2,896 probesets were available for analysis in the ECLIPSE study. As shown in **Fig A in** S1 File, the variance of the *target* gene explained by candidate TFs was high, with an average *r*^2^ of 0.35. Overall, there were 745,943 trios (*target* probe-set, *cis*-eQTL, and candidate TF) to be screened for interaction effects. For each of these trios we performed the standard linear regression on non-rank transformed data (*std*) and inverse-normal rank-transformed data (*rkt*), and we considered for both approaches the HC3 correction of the effect estimate variance to account for heteroscedasticity (*h*3 and *rkt*.*h*3, respectively).

**Fig 5:**
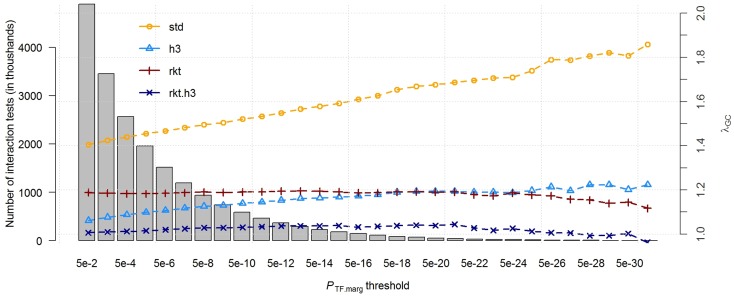
Distribution of interaction test lambdaGC in ECLIPSE. We derived the genomic inflation factor (*λ*_*GC*_) of the standard interaction test using across sub-groups stratified based on P_*TF*.*marg*_, the *p*-value for association between the target gene and the candidate transcription factors (TFs). Grey bars present the total number of interaction tests falling in each strata. Four approaches were performed: i) no normal rank-transformation of the expression data (*std*), ii) HC3 correction of the effect estimate variance to account for heteroscedasticity (*h*3), iii) normal rank-transformation of expression data (*rkt*), and iv) HC3 correction and normal rank-transformation of expression data (*rkt*.*h*3).

**Fig L in** S1 File presents the QQplots observed for each of the four strategies. As in the simulations, we observed non-linear and strong inflation of low *p*-values, as measured by the genomic control (*λ*_*GC*_ equal 1.36, 1.13, 1.22 and 1.10 for *std*, *h*3, *rkt* and *rkt*.*h*3, respectively). There were 151, 244, 4, and 75 significant interactions after correction for multiple comparisons (*P*-value < 1.0x10^-8^), for *std*, *h*3, *rkt* and *rkt*.*h*3, respectively. While a few interactions were significant or near significant across all tests, most showed strong heterogeneity. Table 1 presents the top five interactions from each approach as well as the corresponding *p*-value and rank. Unsurprisingly, all SNPs from Table 1 had a strong marginal effect on the *target* gene. For example, rs8109474 explained 63% of the variance of target probe set 218824_at (*PNMAL1*). The significance of the interaction was also strongly correlated with the strengths of the marginal association between the candidate TF and the *target* probe set. As shown in Fig 5, *λ*_*GC*_ value for the *std* interaction test increases to almost 2 when focusing on the candidate TFs showing the strongest association with the target gene. While some of the observed *λ*_*GC*_ inflation might due to a true enrichment for interaction effects, a substantial part of the association is likely due to statistical artifacts. Conversely, the *rkt* test, which corrects for the effect of outliers and potentially reduces inflation caused by interaction between SNPs and unmeasured factors (**Table A in** S1 File), shows lower inflation (average *λ*_*GC*_ = 1.17). In addition, *rkt* appears to be stable when focusing on TFs with increasing association with the target. Assuming strongly associated TFs are more likely to have biological interaction with cis-eQTL SNPs, this flat curve raises the potential concern that, in agreement with our simulations, interaction effects on the original scale might be removed by the *rkt* transformation.

**Table 1:** Top 5 interaction signals for four different analytical strategies.

SNP	Target	TF	*P*-value (rank*)			
			STD	HC3	RKT	RKT.HC3
rs8109474	*PNMAL1*	*NR3C2*	6.8E-17 *(1)*	2.0E-15 *(7)*	1.3E-05 *(510)*	1.6E-06 *(530)*
rs8109474	*PNMAL1*	*SOX13*	5.2E-16 *(2)*	2.7E-07 *(671)*	1.8E-05 *(630)*	1.5E-04 *(5648)*
rs8109474	*PNMAL1*	*NFIA*	8.2E-15 *(3)*	4.7E-17 *(2)*	3.6E-05 *(1071)*	1.6E-05 *(1668)*
rs8109474	*PNMAL1*	*ZNF30*	4.3E-14 *(4)*	5.1E-15 *(8)*	4.5E-04 *(7844)*	3.9E-04 *(9999)*
rs8109474	*PNMAL1*	*ZBTB7C*	1.3E-13 *(5)*	1.3E-07 *(536)*	3.7E-05 *(1095)*	1.1E-04 *(4692)*
rs364734	*SLC17A5*	*ZFP1*	1.1E-07 *(367)*	7.2E-18 *(1)*	5.9E-08 *(7)*	3.2E-07 *(286)*
rs8109474	*PNMAL1*	*NFIA*	8.2E-15 *(3)*	4.7E-17 *(2)*	3.6E-05 *(1071)*	1.6E-05 *(1668)*
rs62323426	*LINC01091*	*SP2*	1.8E-12 *(16)*	1.7E-16 *(3)*	3.7E-04 *(6763)*	2.9E-06 *(717)*
rs8109474	*PNMAL1*	*ZNF74*	4.0E-10 *(56)*	4.2E-16 *(4)*	6.9E-04 *(11021)*	1.3E-04 *(5187)*
rs8109474	*PNMAL1*	*NFAT5*	1.0E-11 *(23)*	7.2E-16 *(5)*	1.2E-04 *(2678)*	1.1E-06 *(470)*
rs4872978	*LINC00293*	*GATA5*	1.5E-07 *(427)*	1.1E-05 *(2889)*	5.0E-09 *(1)*	1.9E-04 *(6619)*
rs4872978	*LINC00293*	*PAX2*	3.4E-10 *(54)*	0.52 *(2600803)*	6.0E-09 *(2)*	5.8E-09 *(61)*
rs4872978	*LINC00293*	*FOXD3*	2.6E-11 *(26)*	8.5E-11 *(58)*	6.9E-09 *(3)*	2.1E-04 *(7034)*
rs4872978	*LINC00293*	*GATA4*	8.7E-09 *(142)*	2.1E-07 *(628)*	7.6E-09 *(4)*	4.8E-09 *(57)*
rs4872978	*LINC00293*	*DLX6*	4.3E-10 *(57)*	1.6E-08 *(280)*	1.9E-08 *(5)*	1.9E-03 *(27599)*
rs17561351	*PVRL2*	*FOSL2*	2.5E-06 *(1321)*	1.2E-10 *(65)*	5.0E-04 *(8579)*	4.6E-15 *(1)*
rs61001363	*UACA*	*ZNF496*	7.7E-07 *(797)*	5.7E-10 *(103)*	1.9E-06 *(127)*	2.0E-13 *(2)*
rs11983315	*TMEM209*	*FOXN3*	1.0E-05 *(2456)*	6.0E-07 *(938)*	1.7E-04 *(3645)*	1.1E-12 *(3)*
rs254057	*SKP1*	*BCLAF1*	7.8E-04 *(29906)*	2.5E-08 *(326)*	1.2E-03 *(16692)*	2.1E-12 *(4)*
rs7657290	*SCOC*	*TP63*	2.0E-02 *(257900)*	2.3E-10 *(79)*	4.6E-03 *(50506)*	2.3E-12 *(5)*

Abbreviations: STD, standard linear regression; HC3, heteroskedastic variance estimator; RKT, rank-transformed variables; TF, transcription factor.

* Rank of the SNP-target interaction test on the TF over all test performed

## Discussion

Genomic data, and gene-expression data in particular, offer new opportunities to identify gene-gene and gene-environment interactions. In this study we attempted to screen for SNP by TF interaction on gene-expression using data from the ECLIPSE study, and we describe two methodological issues related to the detection and interpretation of statistical genomic interactions. First, interaction effects are scale-dependent and commonly applied pre-processing steps that perform non-linear transformation of expression data can induce or remove statistical interaction, making interpretation of results more challenging. Second, the effect sizes observed in eQTL data are substantially larger than those observed for genetic association with complex human traits and diseases. Assuming interaction effect sizes are similarly large, our simulations show that their presence can induce substantial heteroscedasticity, which itself can impact the robustness of interaction test screening. While heteroscedasticity and scale-dependent effects have been discussed in a broader context, recent screening for interaction effects in gene expression data have not specifically addressed these issues [3, 4, 6]. We used simulation and real data analysis to explore these issues and evaluate the performance of analytical strategies that avoid non-linear transformation of gene expression (*step 3* in Fig 1) while preserving robustness. Our analysis suggests that using the jackknife heteroscedasticity consistent covariance (HC3) correction without applying rank-based inverse normal transformation would be the best approach if sample size is large enough.

Rank-based inverse normal transformation, as well as other non-linear transformations of expression data, can have both positive and negative impact on interaction screening. It removes outliers and assures that the marginal distribution of the phenotype is normal, thus enabling better properties of interaction screening under a complete null model (i.e., in the absence of both main and interaction effect). We also observed in simulated data that heteroscedasticity induced by true interaction effects is partly reduced after applying *rkt* (**Table A in** S1 File). This partially explains the apparent overall better behavior of the *rkt* test in the ECLIPSE data analysis. However, previous work showed that *rkt* is an imperfect solution, because it can impact both type I and type II error rate [21, 26]. Moreover, because it alters the outcome scale, *rkt* can potentially remove the targeted interaction effect and/or induce statistical interaction effects across other SNPs. The question of determining the appropriate scale for interaction testing is a critical issue that remains to be addressed. Rank-based inverse normal transformation or other non-linear transformations that are performed as standard practice for most gene expression analyses fundamentally alter the meaning of statistical interaction in a way that should be explicitly recognized in an interaction analysis. The recent focus on RNA-Seq analysis methods provides an opportunity to revisit this issue. RNA expression is fundamentally heavy-tailed, and many RNA-Seq methods use the negative binomial distribution to model these data, as opposed to the standard quantile normalization and linear modeling approach for microarray data [40]. For the detection of statistical interaction, it is less important to identify a “correct” scale for the analysis, than it is to specify a specific hypothesis for biological interaction and then choose the appropriate scale of the data so that detected statistical interactions will represent biological interactions of interest. From this standpoint, we suggest limiting data transformations and analyzing data on their native scale, though this approach does introduce methodological challenges related to the statistics of non-normal distributions.

Our simulations also demonstrate that an interaction between two factors can induce spurious statistical interaction effects between those factors and other non-interacting factors, leading to the paradoxical situation where the interaction effect screening can be invalid under the scenario of strong interaction. Exploring alternative solutions, we found that interaction effect screening can likely be performed safely among common SNPs (minor allele frequency, MAF ≥ 5%) in large sample sizes (N ≥ 5,000) using the HC3 correction, while for smaller sample size (N ≥ 1000), there will remain uncertainty on the validity of association signal. This result is complementary to previous publications highlighting other challenges in genomic interaction screening. In particular, there has been controversy regarding the validity of previously reported [3] SNP-SNP interactions in eQTL analysis [41, 42]. These studies highlighted that testing for *cis-cis* interaction effects should be interpreted with caution, as an observed statistical interaction may reflect a haplotype effect. While our analysis focused on SNP-by-TF interactions, SNP-SNP interactions would likely face the issues discussed in our study, as the increased type I error rate we observed was driven by the hidden interaction between the SNP tested and an unmeasured quantitative trait, independently of the other tested interacting factor (another SNP in a SNP-SNP interaction screening).

We acknowledge that the proposed strategy does not necessarily represent the optimal solution. To fully address scaling and robustness issues, future work might explore alternatives to the HC3 correction that better model the data (e.g., tests that specifically model residual and predictor distributions) and also assess the impact of common pre-processing practices such as log transformation of raw expression values, quantile normalization [10], adjustment for the principal components of expression [11], and other procedures meant to reduce technical variability [43]. Various combinations of these corrections have been proposed either separately [13] or in an integrated framework [44]. With the exponential increase of genomic data, the validity and performance of existing approaches has been widely discussed for marginal association screening [12, 13, 45–47], and best practices evolve continuously with new methodological developments and the rise of new technologies. While the proposed approach is an important step toward more robust and interpretable interaction effects screening in genomic data, identifying the optimal analytical strategy will similarly follow an iterative process with additional theoretical work and validation from real data applications.

## Material and methods

### Non-linear transformation of expression data

We generated an outcome *Y* as a function of a SNP *G*, an exposure *E* and a covariate *Z* using the following generative model:

$$Y=\gamma_{0}+\gamma_{G}G+\gamma_{E}E+\gamma_{GE}GE+\gamma_{Z}Z+\varepsilon$$ (1)

where *γ*_0_, *γ*_*G*_, *γ*_*E*_, *γ*_*Z*_ and *γ*_*GE*_ are the intercept and the effects of *G*, *E*, *Z* and the interaction effect between *G* and *E*, respectively; and *ε* is the residual. We set the minor allele frequency of *G* at 0.1, and generated *E* using a normal distribution with mean 0 and variance 1. We considered two scenarios. In the first one, *G* and *E* have only main effects, each explaining 40% of the outcome variance, but they do not interact (*γ*_*GE*_ = 0). In the second scenario, only *E* has a main effect (*γ*_*E*_ ≠ 0) and (*γ*_*G*_ = 0), while *G* has no main effect but influences *Y* through its interaction with *E*. The residual *ε* is generated following a right-skewed normal distribution and is scaled so that the variance of *Y* equals 1. For each scenario we plotted *Y* and *rkt*(*Y*) as a function of *E*, where *rkt*() is a rank-based inverse normal transformation function. The *rkt* transformation was performed using the R function *rntransform()* from the GenABEL R package. We then estimated the effect of *E* on both *Y* and *rkt*(*Y*) by genotypic class using standard linear regression.

### Effect of true interaction on interaction screening

To explore the impact of true interaction effects, we then generated a series of data using Eq (1). For simplicity we standardized all predictors to have mean 0 and variance 1. We vary *γ*_*GE*_, the interaction effect between *G* and *E*, so that the variance of the outcome explained by the interaction *τ*_*G*×*E*_ varies from 0 to 30%, where *τ*_*G*×*E*_ = *var*(*γ*_*GE*_*GE*) / *var*(*Y*). Note that *τ*_*G*×*E*_ is similar to the standard definition of variance explained by interaction effects as all predictors are standardized, however this would not be the case for unstandardized predictors [2]. For simplicity we also assumed that, when relevant, the main effects of either *G*, *E*, or *Z* explain the same amount of variance as the interaction effect. We draw *ε*, the residual of *Y*, from a normal distribution with mean 0 and variance scaled so that the unconditional variance of *Y* equals 1. From this model we generated a series of 10,000 independent replicates of 400 individuals and we conducted a test of ${\hat{\beta }}_{GZ}$, the estimated interaction effect between *G* and *Z* from the model:

$$Y\sim \beta_{G}G+\beta_{Z}Z+\beta_{GZ}GZ+\delta$$ (2)

We generated multiple series of data from this model in the absence of main effects (*γ*_*G*_ = *γ*_*E*_ = *γ*_*Z*_ = 0), including a main effect of *G* (*γ*_*G*_ ≠ 0), a main effect of *E* (*γ*_*E*_ ≠ 0), or a main effect of *G* (*γ*_*Z*_ ≠ 0). We first evaluated the relationship between *τ*_*G*×*E*_ and *δ* the residual variance of the outcome from Eq (2), when stratifying by genotypic class. However, to allow for a clearer comparison we also subtracted *ε* from *δ* (see **Supplementary Note**). We then derive for each simulated scenario the type I error rate *α* of *β*_*GZ*_, the interaction tests between *G* and *Z*, at a *p*-value threshold of 0.05, defined as $\left(\Sigma_{N_{s}}p_{GZ}\leq \alpha \right)/N_{s}$, where *N*_*s*_ the number of simulations equals 10,000 and *p*_*GZ*_ is the *p*-value of the interaction effect.

### Correction for statistical tests

We considered standard linear regression and four alternative approaches that can potentially correct for the non-linear effect of the predictor in the interaction tests. First, we used two heteroscedasticity consistent covariance-based approaches. In brief, we used the interaction effect estimate $\left({\hat{\beta }}_{GZ}\right)$ from Eq (2) and derived ${\hat{\sigma }}_{\beta_{GZ}|HC}$, the standard deviation of the interaction term, using HC0 and HC3 formulation using the *vcov()* function from the Sandwich R package. We then derived the Wald test for each updated variance estimates, $\chi_{HC0}={\hat{\beta }}_{GZ}^{2}/{\hat{\sigma }}_{\beta_{GZ}|HC0}^{2}$ and $\chi_{HC1}={\hat{\beta }}_{GZ}^{2}/{\hat{\sigma }}_{\beta_{GZ}|HC3}^{2}$, and their associated *p*-values. The third correction entails using a dummy variable for *Z* instead of the raw continuous coding. The dummy variable, *Z*_*bin*_, equals 0 for values of *Z* smaller than its median and 1 otherwise. The interaction test is then performed by evaluating the term $\beta_{GZ_{bin}}$ from the following model:

$$Y\sim \beta_{G}G+\beta_{Z}Z_{bin}+\beta_{GZ_{bin}}GZ_{bin}+\delta$$ (3)

The last correction entails using a saturated model where the main effects of the interacting factors (here, *G* and *Z*) are modelled using additional terms. Various saturated models can be built. In these analyses we defined a model that includes a main effect of *Z*^2^ and encodes the main effect of the genotype using two dummy variables corresponding to *G* = 1 and *G* = 2. We tested $\beta_{GZ}^{\prime }$, the interaction effect between *Z* and *G*, using its ordinal coding:

$$Y\sim \beta_{G_{1}}G_{1}+\beta_{G_{2}}G_{2}+\beta_{Z}Z+\beta_{Z^{2}}Z^{2}+\beta_{GZ}^{\prime }\;GZ+\delta$$ (4)

We generated a series of 1 million replicates using the model from Eq (1) where *γ* = (*γ*_*G*_, *γ*_*E*,_ *γ*_*GE*,_ *γ*_*Z*_) are randomly sampled from a uniform distribution (0,1). Unless otherwise specified, continuous variables (*E*, *Z* and *ε*) were drawn randomly from a normal distribution or right-skewed normal distribution, both with mean 0 and variance 1. The residual *ε* was further scaled so that the variance of *Y* explained by the simulated predictors in Eq (1) would vary from 0% to 80%. However, to explore the impact of deviation from normality for each of the three quantitative variables (*E*, *Z* and *ε*) we also performed simulations while using a normal distribution only for all replicates. We also compared scenarios where the either the true interacting exposure *E* or the tested interacting exposure *Z* have a main effect against scenarios where they have no main effect. Finally, we varied the sample size from 100 to 5,000 and the frequency of the coded allele for *G* from 0.05 to 0.5. Overall, our simulations covered 96 scenarios.

### The ECLIPSE data

Study participants included 121 COPD cases genotyped as part of the ECLIPSE study [36] using the genome-wide Illumina HumanHap550 BeadChip. Each participant had data on ~6.1 million SNPs, either directly genotyped or imputed using the 1000 Genomes EUR reference panel (March 2010) [48]. Details on quality control assessment, filtering of the SNPs and genotype imputation have been described elsewhere [49, 50]. Gene expression was measured from whole blood samples on the Affymetrix HGU 133Plus 2.0 chip, and eQTL analysis was performed as previously described [8]. Gene expression data were log-transformed and quantile normalized using the RMA function in the “affy” R package [51] (*step1* in Fig 1). We then regressed expression values on age, gender, the first principal components derived from the genotype data on all ECLIPSE participants [52], and the first 13 principal components from gene expression, retaining residuals from this regression for further eQTL analysis (*step2* in Fig 1). Genotype data of all samples used in this study are available in dbGap (phs001252.v1.p1, *in process*). ECLIPSE expression data has been previously submitted to GEO as part of another project (229 samples) with GSE76705. Note that the ECLIPSE dbGaP submission (phs001252.v1.p1, *in process*) will also contain links to the GEO expression data.

## Supporting information

S1 File Text A, Figures A-L, and Table A. (PDF)

## References

[1] Aschard H, Lutz S, Maus B, Duell EJ, Fingerlin TE, Chatterjee N, et al Challenges and opportunities in genome-wide environmental interaction (GWEI) studies. Human genetics. 2012;131(10):1591–613. Epub 2012/07/05. 10.1007/s00439-012-1192-0 22760307 PMC3677711

[2] Aschard H. A perspective on interaction effects in genetic association studies. Genetic epidemiology. 2016;40(8):678–68. 10.1002/gepi.21989 27390122 PMC5132101

[3] Hemani G, Shakhbazov K, Westra HJ, Esko T, Henders AK, McRae AF, et al Detection and replication of epistasis influencing transcription in humans. Nature. 2014;508(7495):249–53. Epub 2014/02/28. 10.1038/nature13005 24572353 PMC3984375

[4] Becker J, Wendland JR, Haenisch B, Nothen MM, Schumacher J. A systematic eQTL study of cis-trans epistasis in 210 HapMap individuals. European journal of human genetics: EJHG. 2012;20(1):97–101. Epub 2011/08/19. 10.1038/ejhg.2011.156 21847142 PMC3234520

[5] Zhang J, Li J, Deng HW. Identifying gene interaction enrichment for gene expression data. PloS one. 2009;4(11):e8064 Epub 2009/12/04. 10.1371/journal.pone.0008064 19956614 PMC2779493

[6] Brown AA, Buil A, Vinuela A, Lappalainen T, Zheng HF, Richards JB, et al Genetic interactions affecting human gene expression identified by variance association mapping. eLife. 2014;3:e01381 Epub 2014/04/29. 10.7554/eLife.01381 24771767 PMC4017648

[7] Carey VJ, Morgan M, Falcon S, Lazarus R, Gentleman R. GGtools: analysis of genetics of gene expression in bioconductor. Bioinformatics. 2007;23(4):522–3. Epub 2006/12/13. 10.1093/bioinformatics/btl628 17158513

[8] Castaldi PJ, Cho MH, Zhou X, Qiu W, McGeachie M, Celli B, et al Genetic control of gene expression at novel and established chronic obstructive pulmonary disease loci. Human molecular genetics. 2015;24(4):1200–10. Epub 2014/10/16. 10.1093/hmg/ddu525 25315895 PMC4806382

[9] Gaffney DJ, Veyrieras JB, Degner JF, Pique-Regi R, Pai AA, Crawford GE, et al Dissecting the regulatory architecture of gene expression QTLs. Genome biology. 2012;13(1):R7 Epub 2012/02/02. 10.1186/gb-2012-13-1-r7 22293038 PMC3334587

[10] Bolstad BM, Irizarry RA, Astrand M, Speed TP. A comparison of normalization methods for high density oligonucleotide array data based on variance and bias. Bioinformatics. 2003;19(2):185–93. Epub 2003/01/23. 12538238 10.1093/bioinformatics/19.2.185

[11] Leek JT, Scharpf RB, Bravo HC, Simcha D, Langmead B, Johnson WE, et al Tackling the widespread and critical impact of batch effects in high-throughput data. Nature reviews Genetics. 2010;11(10):733–9. Epub 2010/09/15. 10.1038/nrg2825 20838408 PMC3880143

[12] Quackenbush J. Microarray data normalization and transformation. Nature genetics. 2002;32 Suppl:496–501. Epub 2002/11/28. 12454644 10.1038/ng1032

[13] Qin S, Kim J, Arafat D, Gibson G. Effect of normalization on statistical and biological interpretation of gene expression profiles. Frontiers in genetics. 2012;3:160 Epub 2012/01/01. 10.3389/fgene.2012.00160 23755061 PMC3668151

[14] Lappalainen T, Sammeth M, Friedlander MR, t Hoen PA, Monlong J, Rivas MA, et al Transcriptome and genome sequencing uncovers functional variation in humans. Nature. 2013;501(7468):506–11. Epub 2013/09/17. 10.1038/nature12531 24037378 PMC3918453

[15] Andersen PK, Skovgaard LT. Multiple regression, the linear predictor. 5 Multiple regression, the linear predictor Statistics for Biology and Health: Springer-Verlag New York; 2010.

[16] Rothman KJ, Greenland S, Lash TL. Chapter 5, Concepts of interaction Modern Epidemiology. 3rd Edition ed: Lippincott Williams & Wilkins; 2012.

[17] Clayton DG. Prediction and interaction in complex disease genetics: experience in type 1 diabetes. PLoS genetics. 2009;5(7):e1000540 Epub 2009/07/09. 10.1371/journal.pgen.1000540 19584936 PMC2703795

[18] Aschard H, Zaitlen N, Tamimi RM, Lindstrom S, Kraft P. A nonparametric test to detect quantitative trait loci where the phenotypic distribution differs by genotypes. Genetic epidemiology. 2013;37(4):323–33. Epub 2013/03/21. 10.1002/gepi.21716 23512279 PMC4088942

[19] Pare G, Cook NR, Ridker PM, Chasman DI. On the use of variance per genotype as a tool to identify quantitative trait interaction effects: a report from the Women's Genome Health Study. PLoS genetics. 2010;6(6):e1000981 Epub 2010/06/30. 10.1371/journal.pgen.1000981 20585554 PMC2887471

[20] Sokal RR, Rohlf FJ. Biometry The Principles and Practice of Statistics in Biological Research. 3rd edition ed: W. H. Freeman; 1994.

[21] Beasley TM, Erickson S, Allison DB. Rank-based inverse normal transformations are increasingly used, but are they merited? Behavior genetics. 2009;39(5):580–95. Epub 2009/06/16. 10.1007/s10519-009-9281-0 19526352 PMC2921808

[22] Xiao X, White EP, Hooten MB, Durham SL. On the use of log-transformation vs. nonlinear regression for analyzing biological power laws. Ecology. 2011;92(10):1887–94. Epub 2011/11/12. 22073779 10.1890/11-0538.1

[23] Navon R, Wang H, Steinfeld I, Tsalenko A, Ben-Dor A, Yakhini Z. Novel rank-based statistical methods reveal microRNAs with differential expression in multiple cancer types. PloS one. 2009;4(11):e8003 Epub 2009/12/01. 10.1371/journal.pone.0008003 19946373 PMC2777376

[24] Breitling R, Herzyk P. Rank-based methods as a non-parametric alternative of the T-statistic for the analysis of biological microarray data. Journal of bioinformatics and computational biology. 2005;3(5):1171–89. Epub 2005/11/10. 16278953 10.1142/s0219720005001442

[25] Rantalainen M, Lindgren CM, Holmes CC. Robust Linear Models for Cis-eQTL Analysis. PloS one. 2015;10(5):e0127882 Epub 2015/05/21. 10.1371/journal.pone.0127882 25992607 PMC4436354

[26] Clifford Blair R, Sawilowsky SS, Higgins JJ. Limitations of the rank transform statistic in tests for interactions. Communications in Statistics—Simulation and Computation. 1987;16(4):1133–45.

[27] White H. A Heteroskedasticity-Consistent Covariance Matrix Estimator and a Direct Test for Heteroskedasticity. Econometrica. 1980;48(4):817–38.

[28] Voorman A, Lumley T, McKnight B, Rice K. Behavior of QQ-plots and genomic control in studies of gene-environment interaction. PloS one. 2011;6(5):e19416 Epub 2011/05/19. 10.1371/journal.pone.0019416 21589913 PMC3093379

[29] Tchetgen Tchetgen EJ, Kraft P. On the robustness of tests of genetic associations incorporating gene-environment interaction when the environmental exposure is misspecified. Epidemiology. 2011;22(2):257–61. Epub 2011/01/14. 10.1097/EDE.0b013e31820877c5 21228699 PMC5972372

[30] Cornelis MC, Tchetgen EJ, Liang L, Qi L, Chatterjee N, Hu FB, et al Gene-environment interactions in genome-wide association studies: a comparative study of tests applied to empirical studies of type 2 diabetes. American journal of epidemiology. 2012;175(3):191–202. Epub 2011/12/27. 10.1093/aje/kwr368 22199026 PMC3261439

[31] MacKinnon J. Thirty Years of Heteroskedasticity-Robust Inference In: Chen X, Swanson NR, editors. Recent Advances and Future Directions in Causality, Prediction, and Specification Analysis: Springer New York; 2013 p. 437–61.

[32] MacKinnon J, White H. Some Heteroskedasticity-consistent Covariance Matrix Estimators with Improved Finite Sample Properties. Journal of Econometrics. 1985;29:305–25.

[33] Almli LM, Duncan R, Feng H, Ghosh D, Binder EB, Bradley B, et al Correcting systematic inflation in genetic association tests that consider interaction effects: application to a genome-wide association study of posttraumatic stress disorder. JAMA psychiatry. 2014;71(12):1392–9. Epub 2014/10/30. 10.1001/jamapsychiatry.2014.1339 25354142 PMC4293022

[34] Aulchenko YS, Struchalin MV, van Duijn CM. ProbABEL package for genome-wide association analysis of imputed data. BMC bioinformatics. 2010;11:134 Epub 2010/03/18. 10.1186/1471-2105-11-134 20233392 PMC2846909

[35] Devlin B, Roeder K. Genomic control for association studies. Biometrics. 1999;55(4):997–1004. Epub 2001/04/21. 11315092 10.1111/j.0006-341x.1999.00997.x

[36] Vestbo J, Anderson W, Coxson HO, Crim C, Dawber F, Edwards L, et al Evaluation of COPD Longitudinally to Identify Predictive Surrogate End-points (ECLIPSE). The European respiratory journal: official journal of the European Society for Clinical Respiratory Physiology. 2008;31(4):869–73. Epub 2008/01/25. 10.1183/09031936.00111707 18216052

[37] Lawrence M, Huber W, Pages H, Aboyoun P, Carlson M, Gentleman R, et al Software for computing and annotating genomic ranges. PLoS computational biology. 2013;9(8):e1003118 Epub 2013/08/21. 10.1371/journal.pcbi.1003118 23950696 PMC3738458

[38] Stranger BE, Nica AC, Forrest MS, Dimas A, Bird CP, Beazley C, et al Population genomics of human gene expression. Nature genetics. 2007;39(10):1217–24. Epub 2007/09/18. 10.1038/ng2142 17873874 PMC2683249

[39] Vaquerizas JM, Kummerfeld SK, Teichmann SA, Luscombe NM. A census of human transcription factors: function, expression and evolution. Nature reviews Genetics. 2009;10(4):252–63. Epub 2009/03/11. 10.1038/nrg2538 19274049

[40] Robinson MD, McCarthy DJ, Smyth GK. edgeR: a Bioconductor package for differential expression analysis of digital gene expression data. Bioinformatics. 2010;26(1):139–40. Epub 2009/11/17. 10.1093/bioinformatics/btp616 19910308 PMC2796818

[41] Wood AR, Tuke MA, Nalls MA, Hernandez DG, Bandinelli S, Singleton AB, et al Another explanation for apparent epistasis. Nature. 2014;514(7520):E3–5. Epub 2014/10/04. 10.1038/nature13691 25279928 PMC6478385

[42] Hemani G, Shakhbazov K, Westra HJ, Esko T, Henders AK, McRae AF, et al Hemani et al. reply. Nature. 2014;514(7520):E5–6. Epub 2014/10/04. 10.1038/nature13692 25279929 PMC4404158

[43] Stegle O, Parts L, Piipari M, Winn J, Durbin R. Using probabilistic estimation of expression residuals (PEER) to obtain increased power and interpretability of gene expression analyses. Nature protocols. 2012;7(3):500–7. Epub 2012/02/22. 10.1038/nprot.2011.457 22343431 PMC3398141

[44] Fusi N, Stegle O, Lawrence ND. Joint modelling of confounding factors and prominent genetic regulators provides increased accuracy in genetical genomics studies. PLoS computational biology. 2012;8(1):e1002330 Epub 2012/01/14. 10.1371/journal.pcbi.1002330 22241974 PMC3252274

[45] Geller SC, Gregg JP, Hagerman P, Rocke DM. Transformation and normalization of oligonucleotide microarray data. Bioinformatics. 2003;19(14):1817–23. Epub 2003/09/27. 14512353 10.1093/bioinformatics/btg245

[46] Hansen KD, Irizarry RA, Wu Z. Removing technical variability in RNA-seq data using conditional quantile normalization. Biostatistics. 2012;13(2):204–16. Epub 2012/01/31. 10.1093/biostatistics/kxr054 22285995 PMC3297825

[47] Qiu X, Wu H, Hu R. The impact of quantile and rank normalization procedures on the testing power of gene differential expression analysis. BMC bioinformatics. 2013;14:124 Epub 2013/04/13. 10.1186/1471-2105-14-124 23578321 PMC3660216

[48] Cho MH, Castaldi PJ, Wan ES, Siedlinski M, Hersh CP, Demeo DL, et al A genome-wide association study of COPD identifies a susceptibility locus on chromosome 19q13. Human molecular genetics. 2012;21(4):947–57. Epub 2011/11/15. 10.1093/hmg/ddr524 22080838 PMC3298111

[49] Shabalin AA. Matrix eQTL: ultra fast eQTL analysis via large matrix operations. Bioinformatics. 2012;28(10):1353–8. Epub 2012/04/12. 10.1093/bioinformatics/bts163 22492648 PMC3348564

[50] Qiu W, Cho MH, Riley JH, Anderson WH, Singh D, Bakke P, et al Genetics of sputum gene expression in chronic obstructive pulmonary disease. PloS one. 2011;6(9):e24395 Epub 2011/09/29. 10.1371/journal.pone.0024395 21949713 PMC3174957

[51] Gautier L, Cope L, Bolstad BM, Irizarry RA. affy—analysis of Affymetrix GeneChip data at the probe level. Bioinformatics. 2004;20(3):307–15. Epub 2004/02/13. 10.1093/bioinformatics/btg405 14960456

[52] Wan ES, Cho MH, Boutaoui N, Klanderman BJ, Sylvia JS, Ziniti JP, et al Genome-wide association analysis of body mass in chronic obstructive pulmonary disease. American journal of respiratory cell and molecular biology. 2011;45(2):304–10. Epub 2010/11/03. 10.1165/rcmb.2010-0294OC 21037115 PMC3266061

