# Supplementary material for: Screening for interaction effects in gene expression data
Source: PLoS One. 2017 Mar 16;12(3):e0173847. doi: 10.1371/journal.pone.0173847 (PMC5354413; doi:10.1371/journal.pone.0173847)

Screening for interaction effects in gene expression data
Supplementary Data

Contents

Text A. Expected variance heterogeneity in the presence of interaction effects 2

Table A. Heterogeneity of variance of the outcome residual by genotype before and after rank-based inverse normal transformation 4

Figure A. Proportion of expression variance explained by SNPs and transcription factors. 5

Figure B. Effect of rank-based inverse normal transformation when residuals are normally distributed. 6

Figure C. Effect of rank-based inverse normal transformation when residuals are non-normally distributed. 7

Figure D. Robustness comparison when Z has a main effect 8

Figure E. Robustness comparison when Z has no main effect 9

Figure F. Robustness comparison when Z has a main effect and is normally distributed 10

Figure G. Robustness comparison when Z has no main effect and is normally distributed 11

Figure H. Robustness comparison when Z has a main effect and E is normally distributed 12

Figure I. Robustness comparison when Z has no main effect and E is normally distributed 13

Figure J. Robustness comparison when Z has a main effect and E is normally distributed 14

Figure K. Robustness comparison when Z has no main effect and E is normally distributed 15

Figure L. QQplot from the ECLIPSE analysis. 16

Text A. Expected variance heterogeneity in the presence of interaction effects

By construction, δ_i, the residual for individual i from equation (B) can be expressed using parameters from equation (A) and (B):

$$\delta_i = Y_i - \left(\hat{\beta}_G G_i + \hat{\beta}_Z Z_i + \hat{\beta}_{GZ} G_i Z_i\right)$$

$$= (\gamma_G G_i + \gamma_E E_i + \gamma_{GE} G_i E_i + \gamma_Z Z_i + \varepsilon_i) - \left(\hat{\beta}_G G_i + \hat{\beta}_Z Z_i + \hat{\beta}_{GZ} G_i Z_i\right)$$

$$= \left(\gamma_G - \hat{\beta}_G\right) G_i + \gamma_E E_i + \gamma_{GE} G_i E_i + \left(\gamma_Z - \hat{\beta}_Z\right) Z_i + \hat{\beta}_{GZ} G_i Z_i + \varepsilon_i$$

It follows that the expected value of δ equals:

$$\mathbb{E}[\delta|G,E,Z] = \mathbb{E}\left[\left(\gamma_G - \hat{\beta}_G\right)G\right] + \mathbb{E}[\gamma_E E] + \mathbb{E}[\gamma_{GE} GE] + \mathbb{E}\left[\left(\gamma_Z - \hat{\beta}_Z\right)Z\right] + \mathbb{E}\left[\hat{\beta}_{GZ} GZ\right] + \mathbb{E}[\varepsilon]$$

$$= \left(\gamma_G - \mathbb{E}\left[\hat{\beta}_G\right]\right)G + \gamma_E E + \gamma_{GE} GE + \left(\gamma_Z - \mathbb{E}\left[\hat{\beta}_Z\right]\right)Z_i + \mathbb{E}\left[\hat{\beta}_{GZ}\right]GZ$$

Assuming ordinary least square estimates are unbiased, which would be true even in the presence of heteroscedasticity, we have $\mathbb{E}\left[\hat{\beta}_Z\right] = \gamma_Z$, $\mathbb{E}\left[\hat{\beta}_{GZ}\right] = 0$, and $\mathbb{E}\left[\hat{\beta}_G\right] = \gamma_G + \gamma_{GE}\mu_E$, and the above equation simplifies to:

$$\mathbb{E}[\delta|G,E,Z] = \mathbb{E}[\delta|G,E] = -\gamma_{GE}\mu_E G + \gamma_E E + \gamma_{GE} GE$$

Assuming G, E, Z and ε are independent, and using the notation $Var(X) = \sigma_X^2$, the variance of δ conditional on G for a sample size of N can then be written as:

$$Var(\delta|G) = Var\left(\left(\gamma_G - \hat{\beta}_G\right)G + \gamma_E E + \gamma_{GE} GE + \left(\gamma_Z - \hat{\beta}_Z\right)Z + \hat{\beta}_{GZ} GZ|G\right) + \varepsilon$$

$$= Var\left(\hat{\beta}_G\right)G^2 + (\gamma_E + \gamma_{GE} G)^2 \sigma_E^2 + Var\left(\left(\gamma_Z - \hat{\beta}_Z\right)Z\right) + Var\left(\hat{\beta}_{GZ} Z\right)G^2 + \sigma_\varepsilon^2$$

We derive the three unsolved variance terms:

$$Var\left(\hat{\beta}_G\right) = \frac{\sigma_\varepsilon^2}{N \times \sigma_G^2}$$

$$Var\left(\left(\gamma_Z - \hat{\beta}_Z\right)Z\right) = \mathbb{E}\left[\left(\gamma_Z - \hat{\beta}_Z\right)^2\right]\mathbb{E}[Z^2] - \mathbb{E}\left[\gamma_Z - \hat{\beta}_Z\right]^2 \mathbb{E}[Z]^2$$

$$= \left(\mathbb{E}[\gamma_Z^2] + \mathbb{E}\left[\hat{\beta}_Z^2\right] - \mathbb{E}\left[2\gamma_Z \hat{\beta}_Z\right]\right)\mathbb{E}[Z^2]$$

$$= \left(\mathbb{E}[\gamma_Z^2] + \left(\mathbb{E}\left[\hat{\beta}_Z\right]^2 + \frac{\sigma_\varepsilon^2}{N \times \sigma_Z^2}\right) - 2\gamma_Z \mathbb{E}\left[\hat{\beta}_Z\right]\right)\sigma_Z^2$$

$$= \left(\gamma_Z^2 + \left(\gamma_Z^2 + \frac{\sigma_\varepsilon^2}{N \times \sigma_Z^2}\right) - 2\gamma_Z^2\right)\sigma_Z^2$$

$$= \frac{\sigma_\varepsilon^2}{N}$$

$$
\begin{aligned}
Var(\hat{\beta}_{GZ}Z) &= \mathbb{E}[\hat{\beta}_{GZ}^2]\mathbb{E}[Z^2] - \mathbb{E}[\hat{\beta}_{GZ}]^2\mathbb{E}[Z]^2 \\
&= \left(\mathbb{E}[\hat{\beta}_{GZ}]^2 + \frac{\sigma_\varepsilon^2}{N \times \sigma_{GZ}^2}\right)\sigma_Z^2 \\
&= \frac{\sigma_\varepsilon^2 \sigma_Z^2}{N \times \sigma_{GZ}^2}
\end{aligned}
$$

It follows that when N is large so that $\frac{1}{N} \ll 1$:

$$Var(\delta|G) \approx (\gamma_E + \gamma_{GE}G)^2\sigma_E^2 + \sigma_\varepsilon^2$$

and,

$$Var(\delta|G = 0) \approx \gamma_E^2\sigma_E^2 + \sigma_\varepsilon^2$$

$$
\begin{aligned}
Var(\delta|G = 1) &\approx (\gamma_E + \gamma_{GE})^2\sigma_E^2 + \sigma_\varepsilon^2 \\
&\approx Var(\delta|G = 0) + (2\gamma_E\gamma_{GE} + \gamma_{GE}^2)\sigma_E^2
\end{aligned}
$$

$$
\begin{aligned}
Var(\delta|G = 2) &\approx (\gamma_E + 2\gamma_{GE})^2\sigma_E^2 + \sigma_\varepsilon^2 \\
&\approx Var(\delta|G = 0) + 4(\gamma_E\gamma_{GE} + \gamma_{GE}^2)\sigma_E^2
\end{aligned}
$$

Thus, γ_{GE} is the sole factor inducing heterogeneity of variance of δ, but the magnitude of the heterogeneity can decrease or increase when $\gamma_E \neq 0$. Importantly, the magnitude of main effect of G and Z can also impact the magnitude of the heterogeneity by reducing σ_ε^2, the residual variance from equation (A), which acts as a diluting factor (e.g. $\sigma_\varepsilon^2 \gg (\gamma_E\gamma_{GE} + \gamma_{GE}^2)\ \sigma_E^2 \Rightarrow Var(\delta_i|G_i = 0) \approx Var(\delta_i|G_i = 1) \approx Var(\delta_i|G_i = 2)$).

Table A. Heterogeneity of variance of the outcome residual by genotype before and after rank-based inverse normal transformation

γ_{GE}^2	***Non-transformed outcome***[a]				***Transformed outcome***[b]			
	$Var(\delta\|G=0)$	$Var(\delta\|G=1)$	$Var(\delta\|G=2)$	P_{lev}*	$Var(\delta\|G=0)$	$Var(\delta\|G=1)$	$Var(\delta\|G=2)$	P_{lev}*
0.000	0.99	0.97	0.96	0.511	1.00	0.98	0.97	0.54
0.020	0.91	0.97	1.03	0.433	0.92	0.97	1.07	0.49
0.040	0.83	0.94	1.24	0.198	0.84	0.93	1.14	0.27
0.060	0.76	0.91	1.27	0.068	0.78	0.91	1.23	0.122
0.080	0.68	0.88	1.46	0.0043	0.70	0.89	1.35	0.020
0.100	0.60	0.86	1.51	6.0E-04	0.65	0.85	1.34	0.011
0.120	0.52	0.83	1.63	9.4E-06	0.58	0.83	1.40	8.9E-04
0.140	0.44	0.80	1.77	4.9E-08	0.51	0.80	1.44	7.3E-05
0.160	0.36	0.78	1.87	9.5E-11	0.44	0.78	1.43	1.9E-06
0.180	0.28	0.76	1.98	2.7E-15	0.36	0.77	1.51	1.9E-08
0.200	0.20	0.73	2.07	8.8E-21	0.28	0.75	1.58	3.2E-11

Data were simulated as described in Figure 3. An outcome Y is simulated as a function of a SNP G, an unmeasured exposure E, a measured exposure Z and a $G \times E$ interaction effect across a series of 10,000 replicates, each including 400 individuals. For simplicity we assumed all predictors have the same effect, i.e. $\gamma_G = \gamma_E = \gamma_Z = \gamma_{GE}$ We varied γ_{GE} from 0 to 0.2, so that total variance of Y explained by all predictors range from 0% to 80% For each value of γ_{GE}, we derived $Var(\delta|G)$, the variance of δ, the residual from equation (B), conditional on G.

[a] *$Var(\delta|G)$ is derived as the median across all replicates for the simulated outcome*

[b] *$Var(\delta|G)$ is derived as the median across all replicates when applying equation (B) after a rank-based inverse normal transformation of the outcome Y.*

The p-value for the heterogeneity of variance (P_{lev}) is derived using the Levene's test.

Figure A. Proportion of expression variance explained by SNPs and transcription factors.

Observed distribution of variance of gene expression explained (r^2, blue bars) for the 3,334 probesets with at least one genome-wide significant *cis*-eQTL SNP (*q*-value <=0.01). Panel (a) shows the r^2 for the corresponding *cis*-eQTL SNPs obtained from marginal genetic models, and panel (b) the r^2 from marginal transcription factor (TF) models for the subset of TFs showing significant association with the probesets after Bonferroni correction.

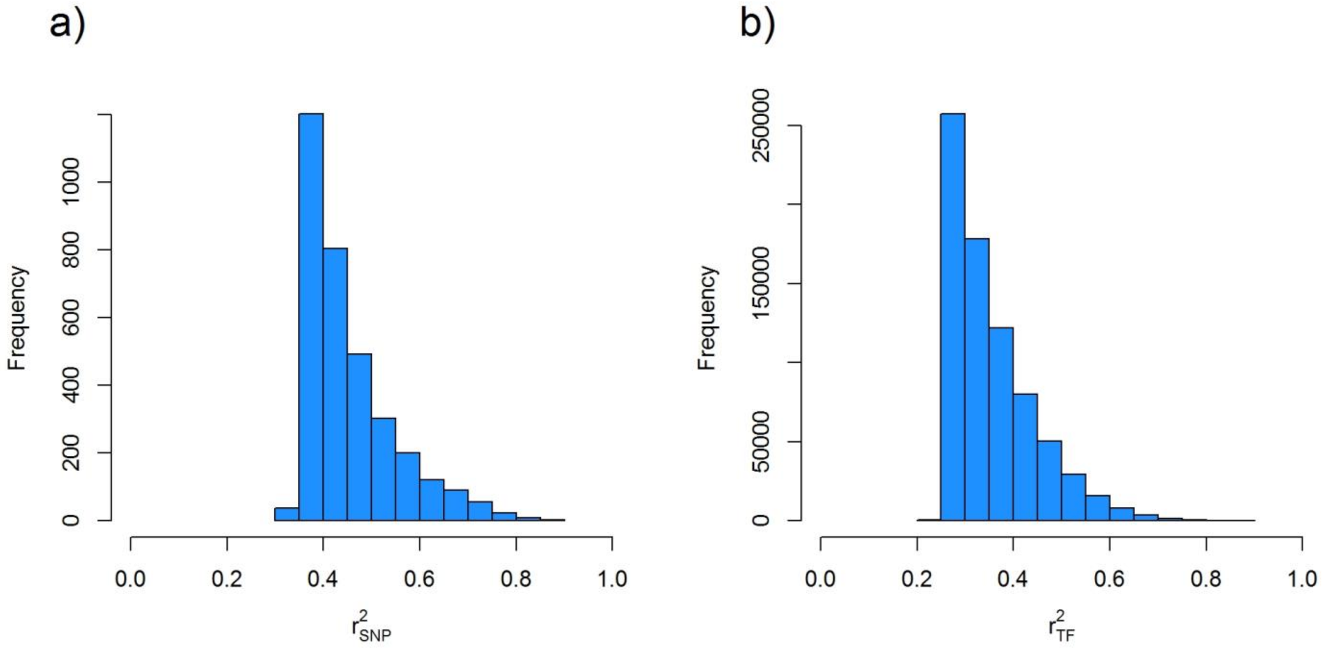

Figure B. Effect of rank-based inverse normal transformation when residuals are normally distributed.

We defined an outcome Y as a function of a single nucleotide polymorphism G with a minor allele frequency of 0.1, an exposure E normally distributed with mean 5 and variance 1, and a normally distributed residual term ε. For illustration purposes we simulated a strong effect, with both G and E each explaining 40% of the variance of Y, but no interaction effect between G and E. Using this model we generated datasets of 100,000 individuals. In a), we plotted the bin counts (upper plots) and density (middle plots) of Y by genotypic classes, and the scatter plot of Y as a function of E, also by genotypic classes (lower plots, trends are indicated by solid lines). Note that we plotted $E \sim Y$ and not the inverse $Y \sim E$ in order to keep the correspondence between all plots. Panel b) shows the same plots after applying a rank-based inverse normal transformation of Y. It appears that the transformation is compressing Y values unevenly by genotypic classes, inducing differences in the variance of Y conditional on G, and resulting in an interaction effect between E and G.

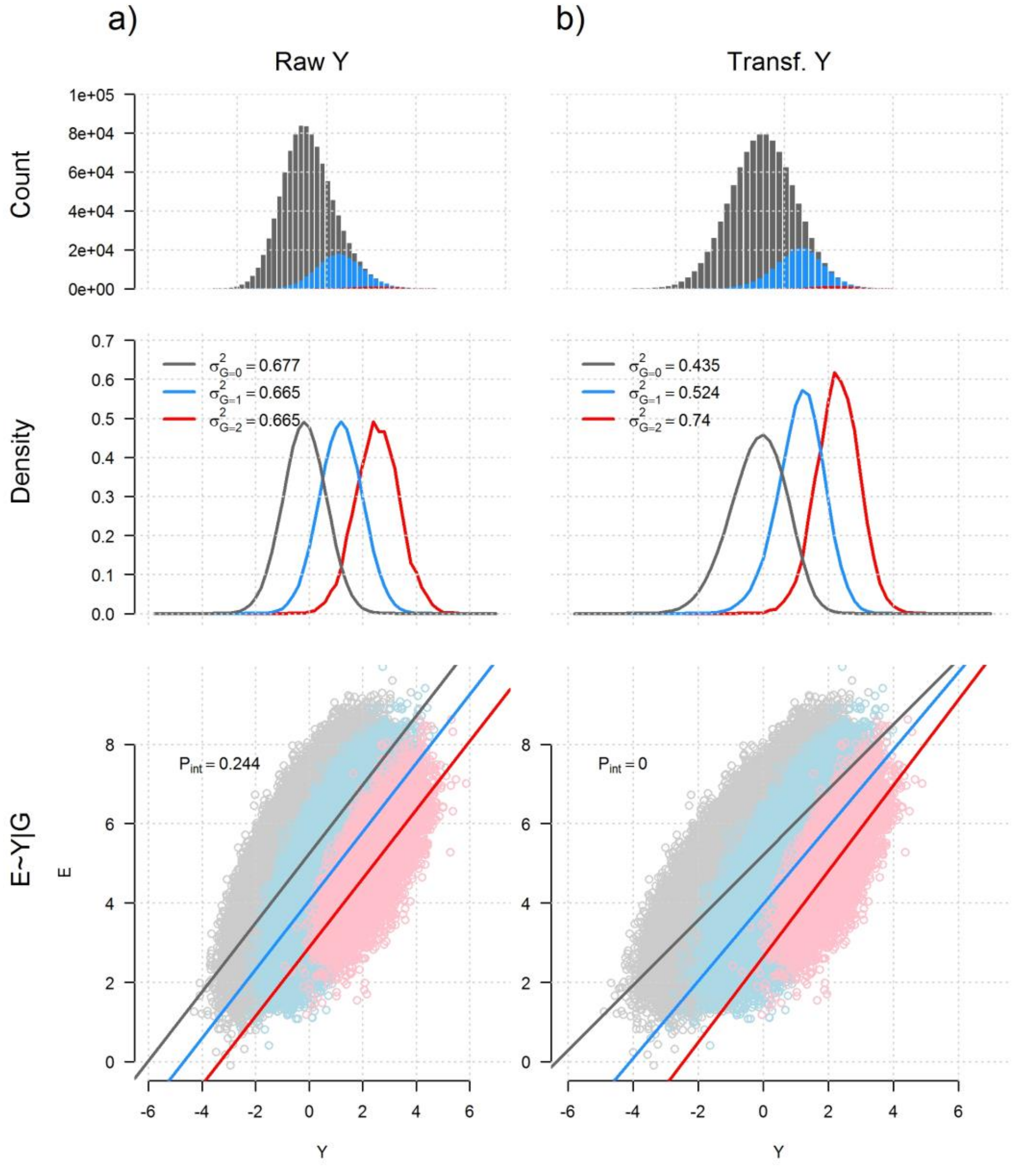

Figure C. Effect of rank-based inverse normal transformation when residuals are non-normally distributed.

We defined an outcome Y as a function of a single nucleotide polymorphism G with a minor allele frequency of 0.1, an exposure E normally distributed with mean 5 and variance 1, and a right-skewed normal distributed residual term ε. For illustration purposes we simulated a strong effect, with both G and E each explaining 40% of the variance of Y with no interaction between G and E. Using this model we generated datasets of 100,000 individuals. In a), we plotted the bin counts (upper plots) and density (middle plots) of Y by genotypic classes, and the scatter plot of Y as a function of E, also by genotypic classes (lower plots, trends are indicated by solid lines). Note that we plotted $E{\sim}Y$ and not the inverse $Y{\sim}E$ in order to keep the correspondence between all plots. Panel b) shows the same plots after applying a rank-based inverse normal transformation of Y. It appears that the transformation is compressing Y values unevenly by genotypic classes, inducing differences in the variance of Y conditional on G, and resulting in an interaction effect between E and G.

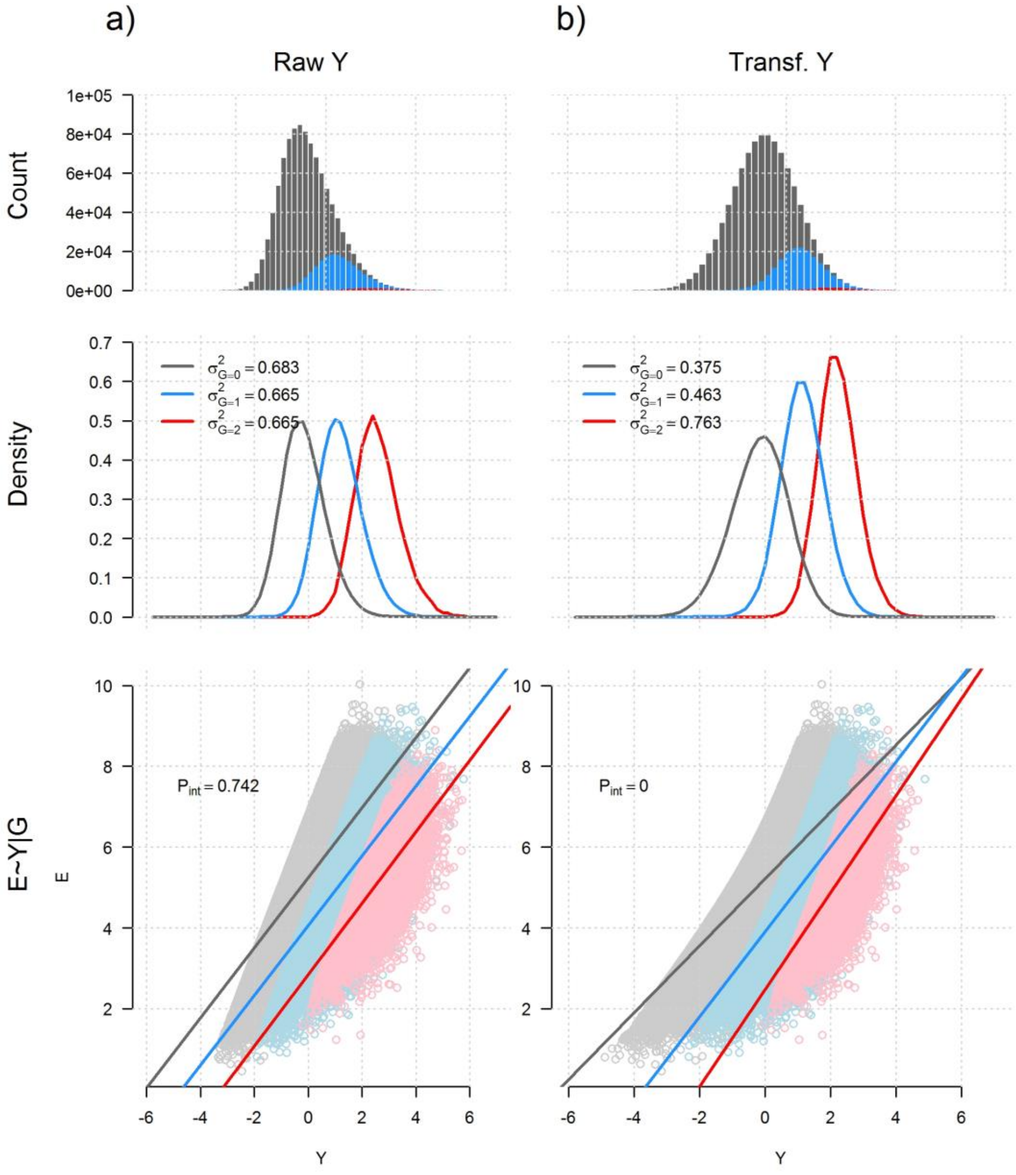

Figure D. Robustness comparison when Z has a main effect

QQplots over a series of 100,000 replicates where an outcome Y is simulated as a function of a genetic variant G, an unmeasured exposure E, an interaction between G and E, and a measured exposure Z. The validity of five tests is evaluated by comparing the observed -log$_{10}$ (p-value) against the expected -log$_{10}$ (p-value) when testing for the null interaction between G and Z. The tests include a standard linear regression using main and interaction terms only (STD), heteroscedasticity consistent-based tests using effect estimates from STD (HC0 and HC3), linear regression using binary-transformed Z (BIN), and a saturated model including a main effect of Z^2 and each genotype coded as dummy variables (SAT). We considered coded allele frequencies (CAF) of 0.05 (first row), 0.3 (middle row) and 0.5 (bottom row), and sample size N of 100, 500, 1,000 and 5,000. We randomly draw E, Z, and ε, the residual of Y from either a normal or a right-skewed normal distribution. For each scenario we derived the genomic inflation factor λ_{GC}.

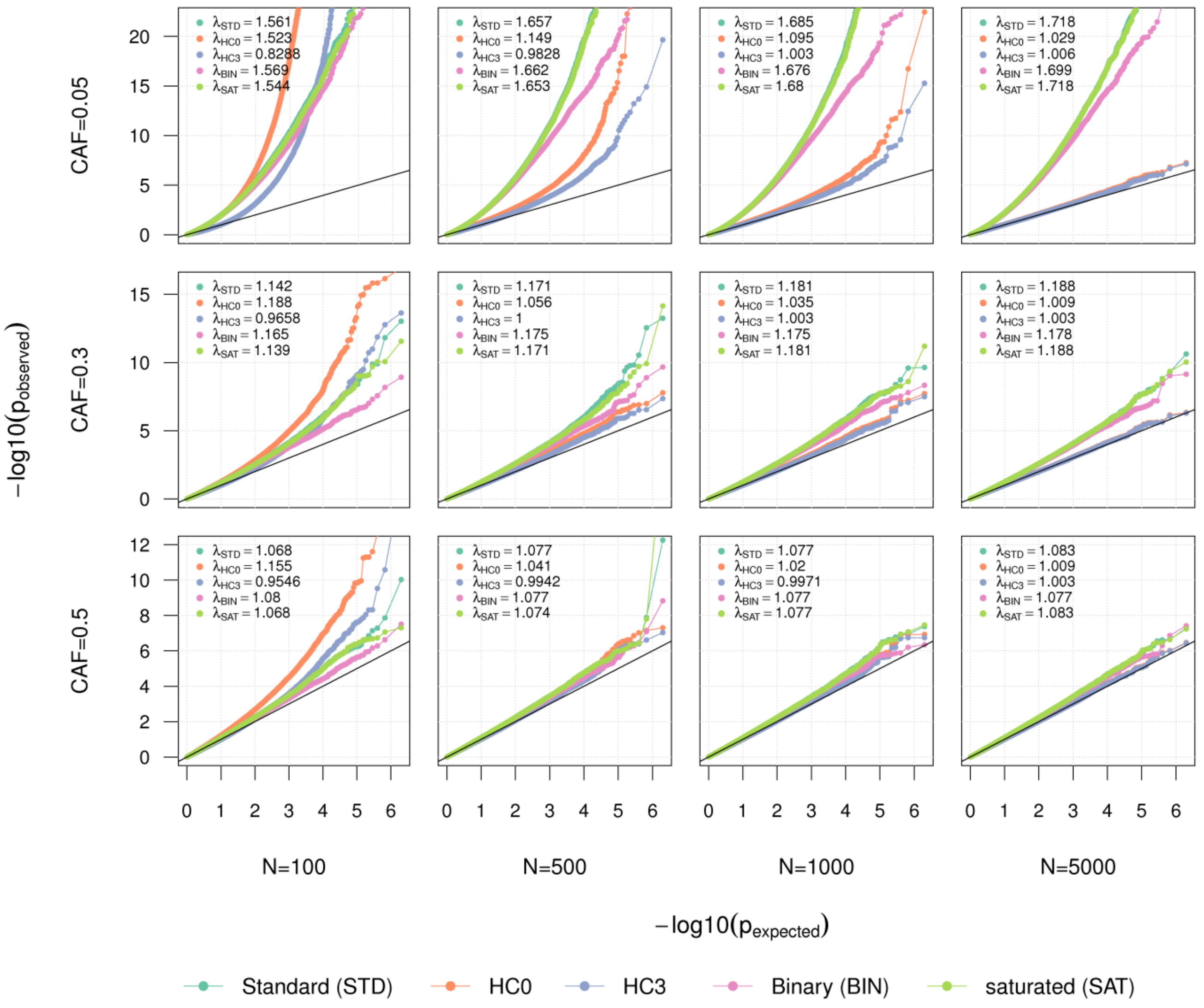

Figure E. Robustness comparison when Z has no main effect

QQplots over a series of 100,000 replicates where an outcome Y is simulated as a function of a genetic variant G, an unmeasured exposure E, an interaction between G and E, but in the absence of main effect of the measured exposure Z. The validity of five tests is evaluated by comparing the observed -$\log_{10}$ (p-value) against the expected -$\log_{10}$ (p-value) when testing for the null interaction between G and Z. The tests include a standard linear regression using main and interaction terms only (STD), heteroscedasticity consistent-based tests using effect estimates from STD (HC0 and HC3), linear regression using binary-transformed Z (BIN), and a saturated model including a main effect of Z^2 and each genotype coded as dummy variables (SAT). We considered coded allele frequencies (CAF) of 0.05 (first row), 0.3 (middle row) and 0.5 (bottom row), and sample size N of 100, 500, 1,000 and 5,000. We randomly draw E, Z, and ε, the residual of Y from either a normal or a right-skewed normal distribution. For each scenario we derived the genomic inflation factor λ_{GC}.

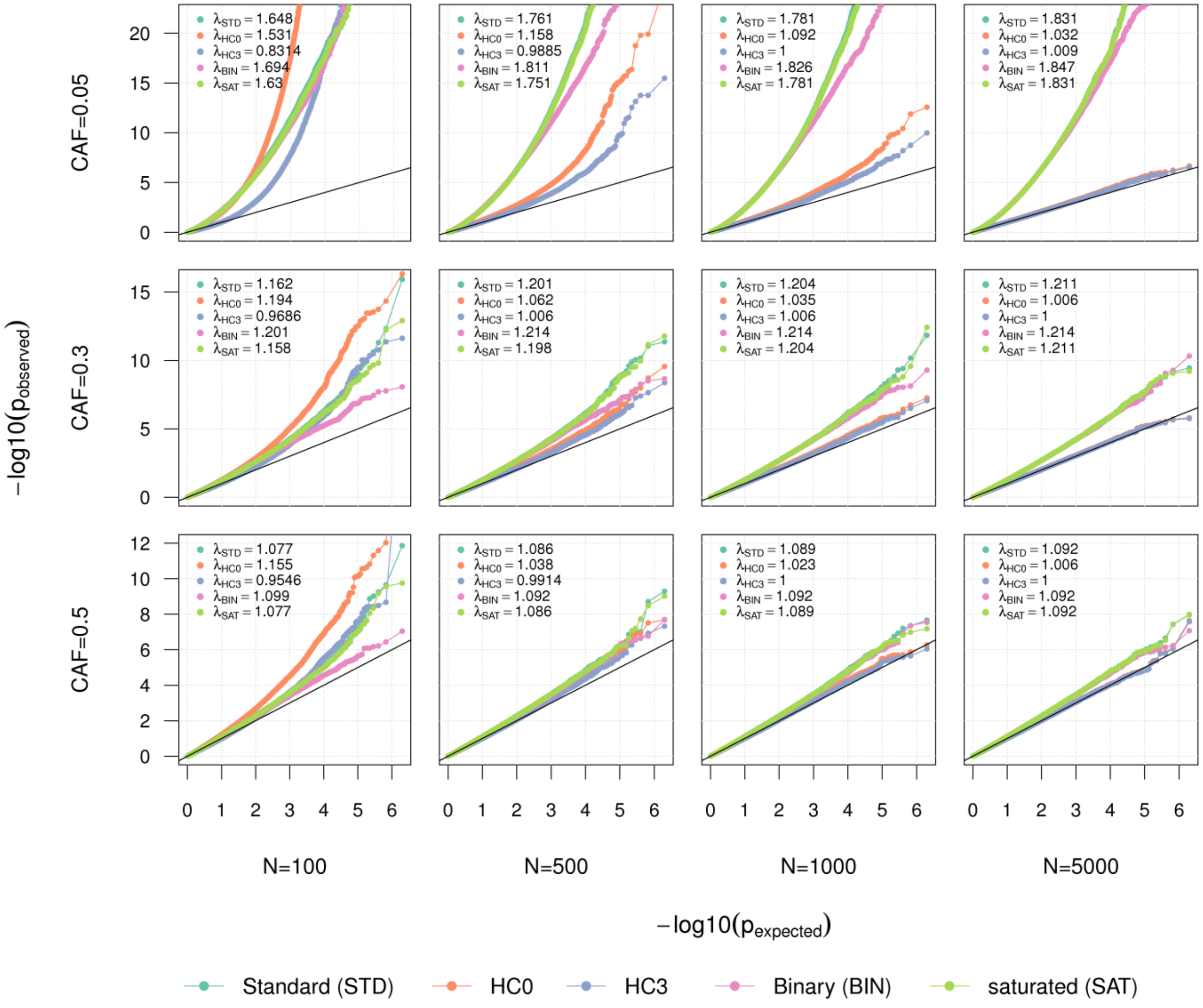

Figure F. Robustness comparison when Z has a main effect and is normally distributed

QQplots over a series of 100,000 replicates where an outcome Y is simulated as a function of a genetic variant G, an unmeasured exposure E, an interaction between G and E, and a measured exposure Z. The validity of five tests is evaluated by comparing the observed -log$_{10}$ (p-value) against the expected -log$_{10}$ (p-value) when testing for the null interaction between G and Z. The tests include a standard linear regression using main and interaction terms only (STD), heteroscedasticity consistent-based tests using effect estimates from STD (HC0 and HC3), linear regression using binary-transformed Z (BIN), and a saturated model including a main effect of Z^2 and each genotype coded as dummy variables (SAT). We considered coded allele frequencies (CAF) of 0.05 (first row), 0.3 (middle row) and 0.5 (bottom row), and sample size N of 100, 500, 1,000 and 5,000. We randomly draw E, and ε, the residual of Y from either a normal or a right-skewed normal distribution, while we generated Z from normal in all replicates. For each scenario we derived the genomic inflation factor λ_{GC}.

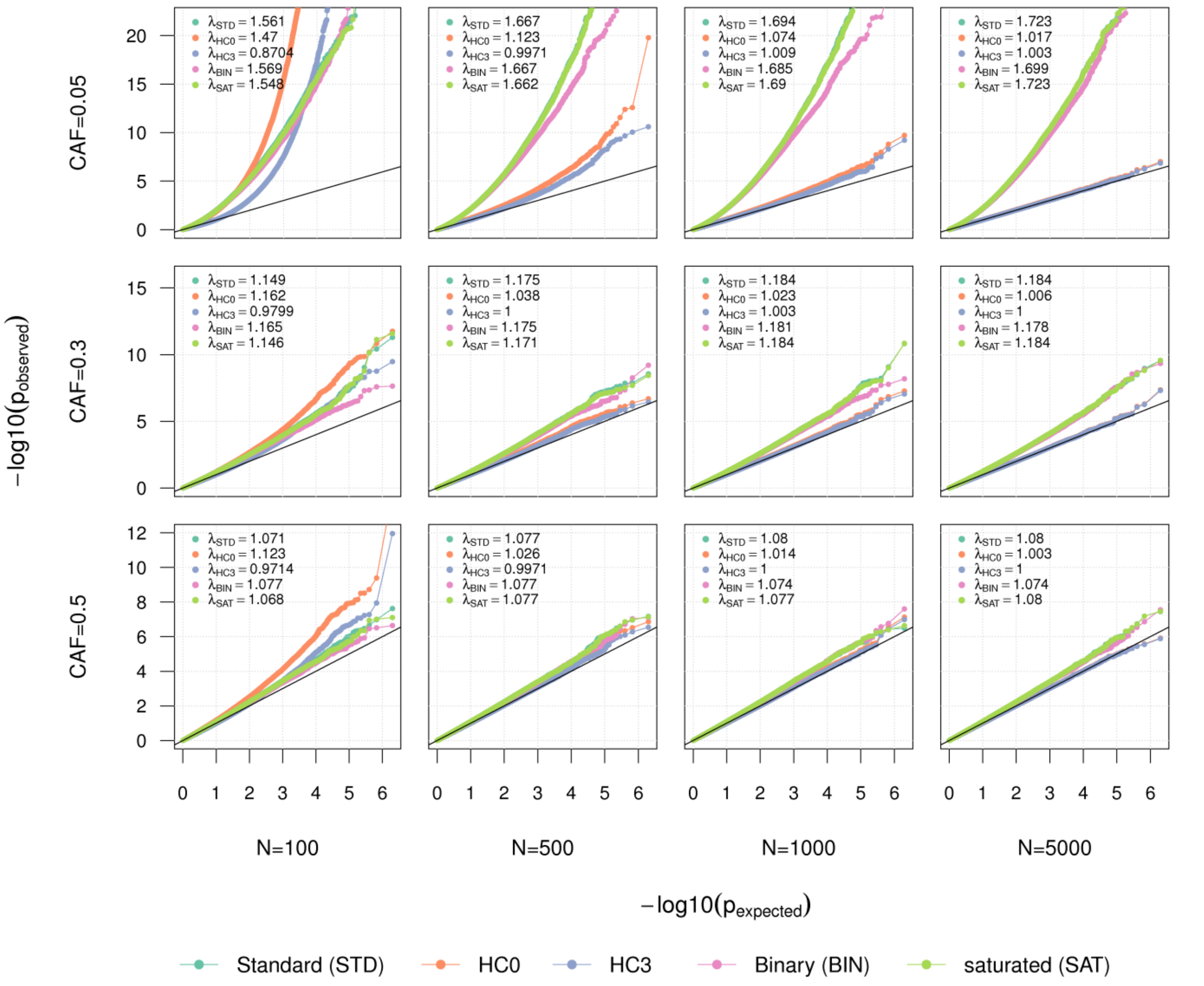

Figure G. Robustness comparison when Z has no main effect and is normally distributed

QQplots over a series of 100,000 replicates where an outcome Y is simulated as a function of a genetic variant G, an unmeasured exposure E, an interaction between G and E, but in the absence of main effect of the measured exposure Z. The validity of five tests is evaluated by comparing the observed -$\log_{10}$ (p-value) against the expected -$\log_{10}$ (p-value) when testing for the null interaction between G and Z. The tests include a standard linear regression using main and interaction terms only (STD), heteroscedasticity consistent-based tests using effect estimates from STD (HC0 and HC3), linear regression using binary-transformed Z (BIN), and a saturated model including a main effect of Z^2 and each genotype coded as dummy variables (SAT). We considered coded allele frequencies (CAF) of 0.05 (first row), 0.3 (middle row) and 0.5 (bottom row), and sample size N of 100, 500, 1,000 and 5,000. We randomly draw E, and ε, the residual of Y from either a normal or a right-skewed normal distribution, while we generated Z from normal in all replicates. For each scenario we derived the genomic inflation factor λ_{GC}.

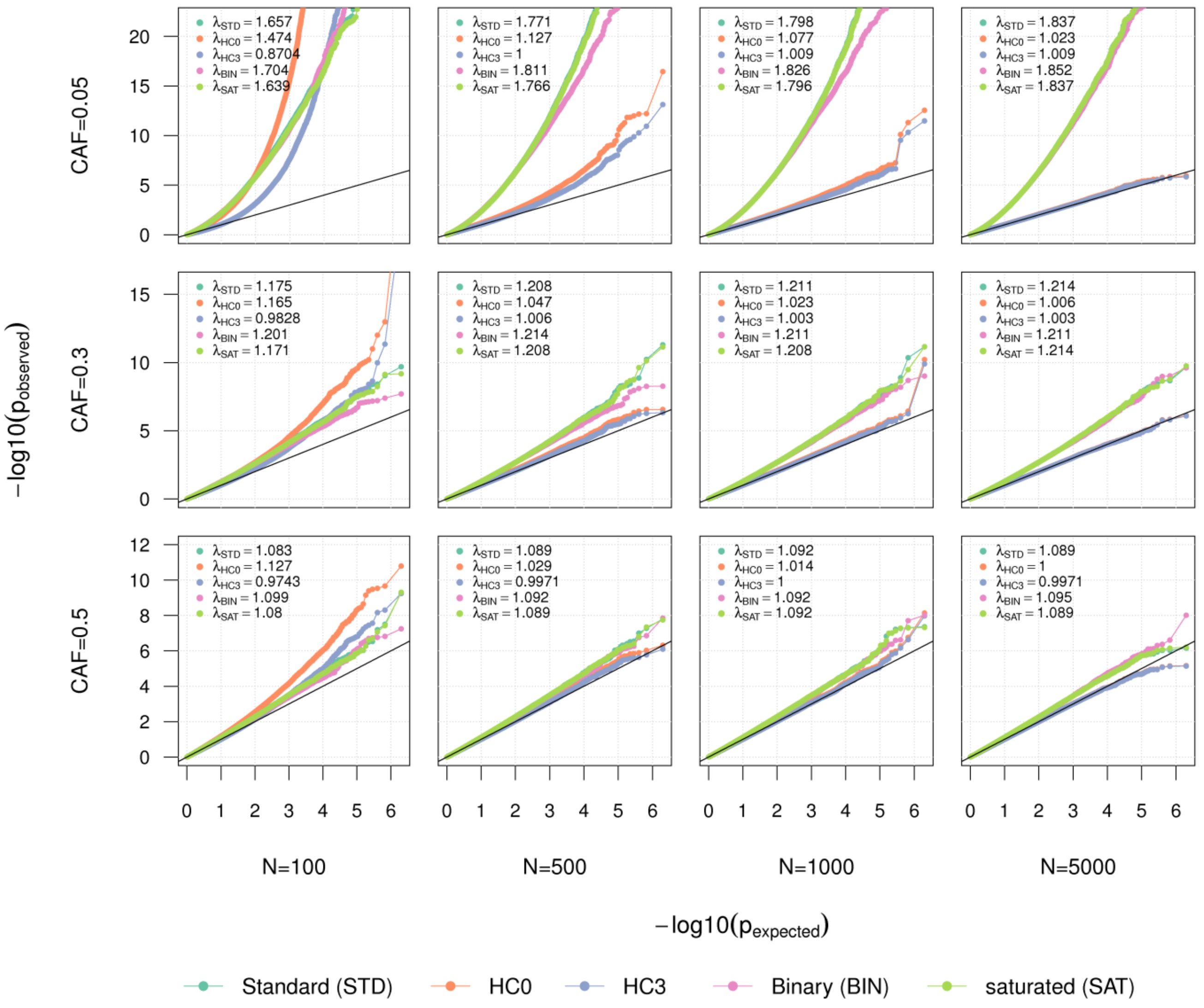

Figure H. Robustness comparison when Z has a main effect and E is normally distributed

QQplots over a series of 100,000 replicates where an outcome Y is simulated as a function of a genetic variant G, an unmeasured exposure E, an interaction between G and E, and a measured exposure Z. The validity of five tests is evaluated by comparing the observed -log$_{10}$ (p-value) against the expected -log$_{10}$ (p-value) when testing for the null interaction between G and Z. The tests include a standard linear regression using main and interaction terms only (STD), heteroscedasticity consistent-based tests using effect estimates from STD (HC0 and HC3), linear regression using binary-transformed Z (BIN), and a saturated model including a main effect of Z^2 and each genotype coded as dummy variables (SAT). We considered coded allele frequency of 0.05 (first row), 0.3 (middle row) and 0.5 (bottom row), and sample size N of 100, 500, 1,000 and 5,000. We randomly draw Z, and ε, the residual of Y from either a normal or a right-skewed normal distribution, while we generated E from normal in all replicates. For each scenario we derived the genomic inflation factor λ_{GC}.

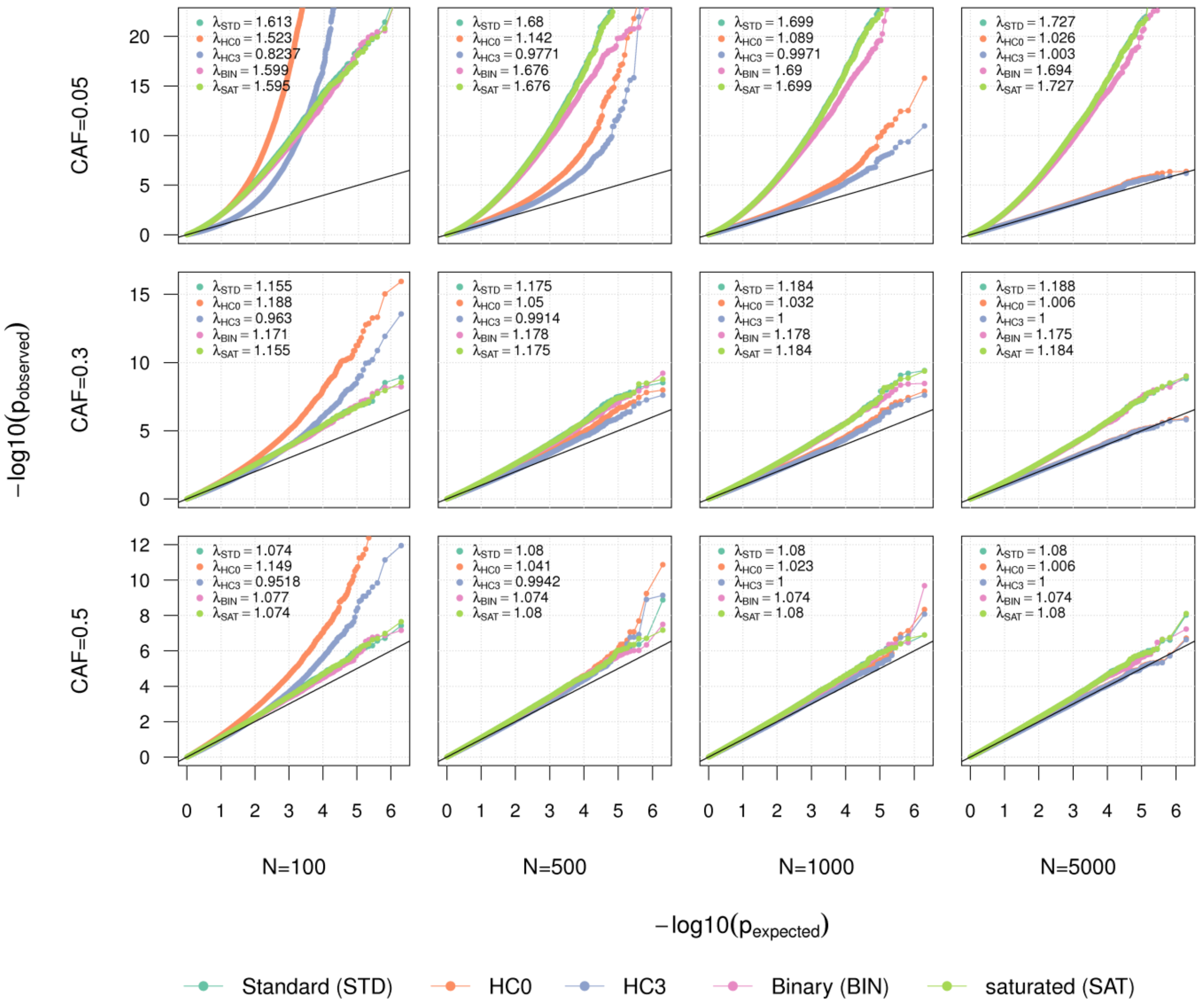

Figure I. Robustness comparison when Z has no main effect and E is normally distributed

QQplots over a series of 100,000 replicates where an outcome Y is simulated as a function of a genetic variant G, an unmeasured exposure E, an interaction between G and E, but in the absence of main effect of the measured exposure Z. The validity of five tests is evaluated by comparing the observed -log$_{10}$ (p-value) against the expected -log$_{10}$ (p-value) when testing for the null interaction between G and Z. The tests include a standard linear regression using main and interaction terms only (STD), heteroscedasticity consistent-based tests using effect estimates from STD (HC0 and HC3), linear regression using binary-transformed Z (BIN), and a saturated model including a main effect of Z^2 and each genotype coded as dummy variables (SAT). We considered coded allele frequencies (CAF) of 0.05 (first row), 0.3 (middle row) and 0.5 (bottom row), and sample size N of 100, 500, 1,000 and 5,000. We randomly draw Z, and ε, the residual of Y from either a normal or a right-skewed normal distribution, while we generated E from normal in all replicates. For each scenario we derived the genomic inflation factor λ_{GC}.

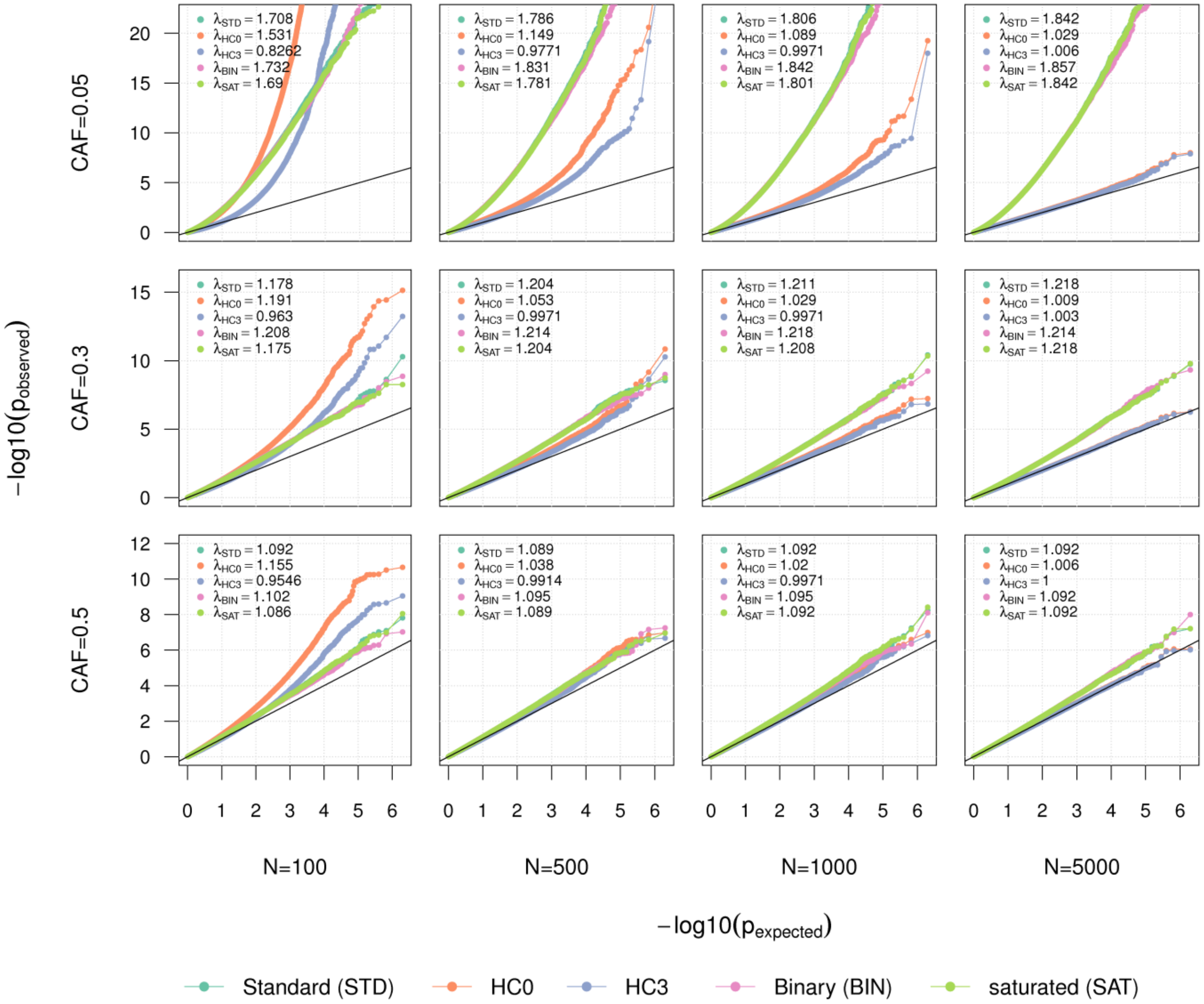

Figure J. Robustness comparison when Z has a main effect and E is normally distributed

QQplots over a series of 100,000 replicates where an outcome Y is simulated as a function of a genetic variant G, an unmeasured exposure E, an interaction between G and E, and a measured exposure Z. The validity of five tests is evaluated by comparing the observed -log$_{10}$ (p-value) against the expected -log$_{10}$ (p-value) when testing for the null interaction between G and Z. The tests include a standard linear regression using main and interaction terms only (STD), heteroscedasticity consistent-based tests using effect estimates from STD (HC0 and HC3), linear regression using binary-transformed Z (BIN), and a saturated model including a main effect of Z^2 and each genotype coded as dummy variables (SAT). We considered coded allele frequencies (CAF) of 0.05 (first row), 0.3 (middle row) and 0.5 (bottom row), and sample size N of 100, 500, 1,000 and 5,000. We randomly draw Z, and E, the residual of Y from either a normal or a right-skewed normal distribution, while we generated ε from normal in all replicates. For each scenario we derived the genomic inflation factor λ_{GC}.

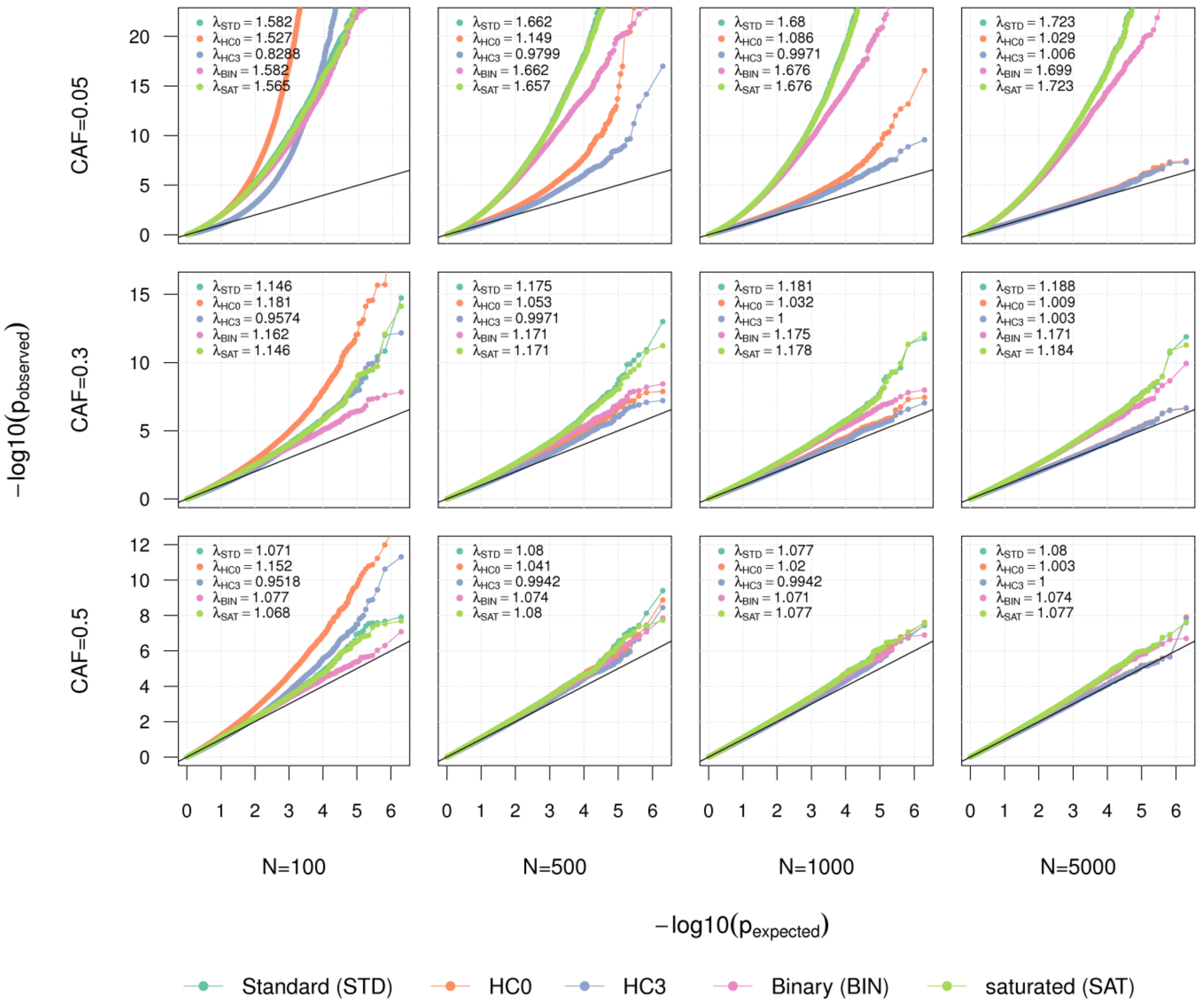

Figure K. Robustness comparison when Z has no main effect and E is normally distributed

QQplots over series of 100,000 replicates where an outcome Y is simulated as a function of a genetic variant G, an unmeasured exposure E, an interaction between G and E, but in the absence of main effect of the measured exposure Z. The validity of five tests is evaluated by comparing the observed -$\log_{10}$ (p-value) against the expected -$\log_{10}$ (p-value) when testing for the null interaction between G and Z. The tests include a standard linear regression using main and interaction terms only (STD), heteroscedasticity consistent-based tests using effect estimates from STD (HC0 and HC3), linear regression using binary-transformed Z (BIN), and a saturated model including a main effect of Z^2 and each genotype coded as dummy variables (SAT). We considered coded allele frequencies (CAF) of 0.05 (first row), 0.3 (middle row) and 0.5 (bottom row), and sample size N of 100, 500, 1,000 and 5,000. We randomly draw Z, and E, the residual of Y from either a normal or a right-skewed normal distribution, while we generated ε from normal in all replicates. For each scenario we derived the genomic inflation factor λ_{GC}.

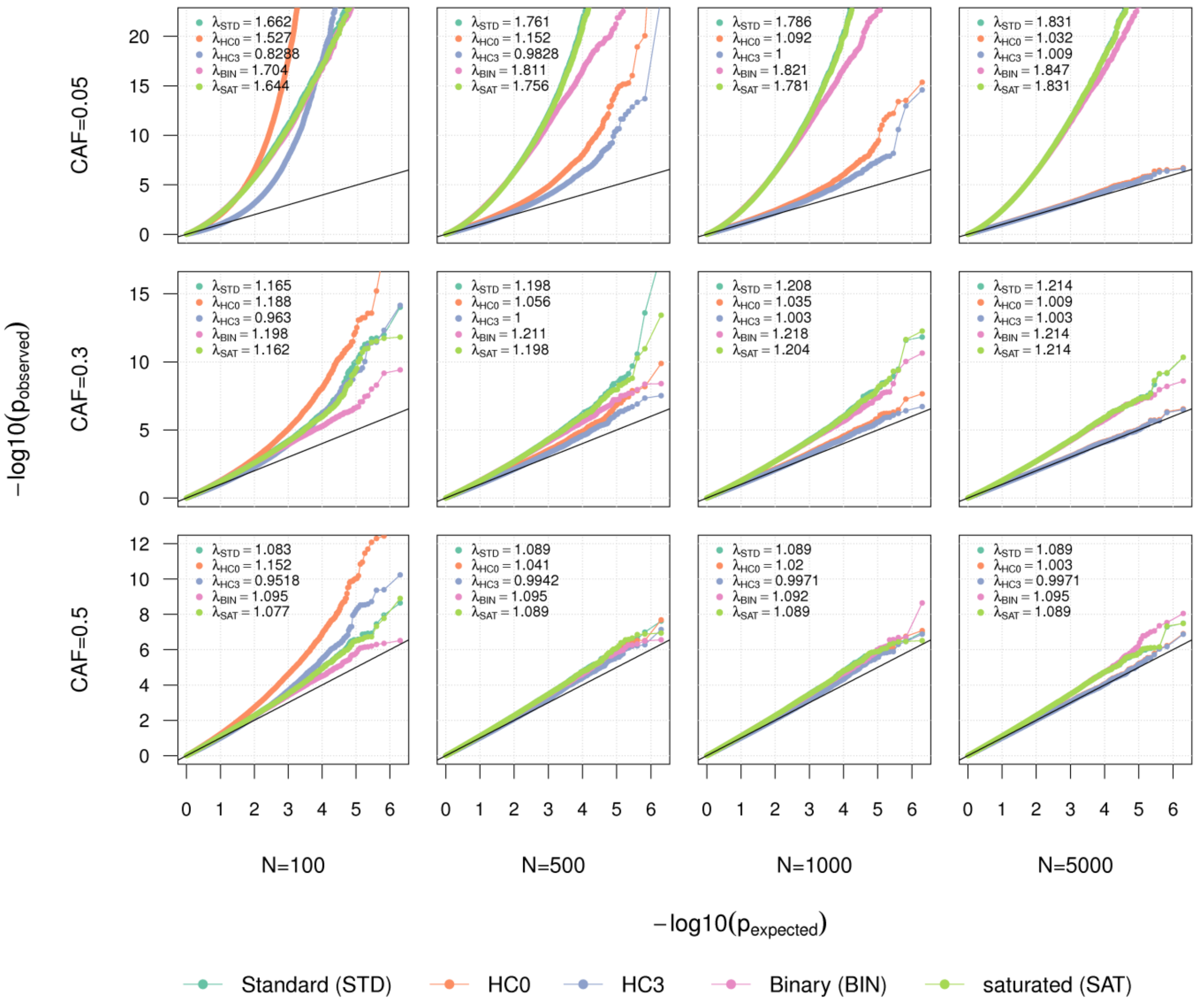

Figure L. QQplot from the ECLIPSE analysis.

QQplots from *cis*-eQTL by transcription factor (TF) interaction effect screening performed over 3,334 probe-sets with at least one significant *cis*-eQTL SNP (q-value <=0.01). Four approaches were performed: i) no normal rank-transformation of the expression data (std), ii) HC3 correction of the effect estimate variance to account for heteroscedasticity ($h3$), iii) normal rank-transformation of expression data (rkt), and iv) HC3 correction and normal rank-transformation of expression data ($rkt.h3$). For each test we derive the genomic inflation factor (λ_{GC}).

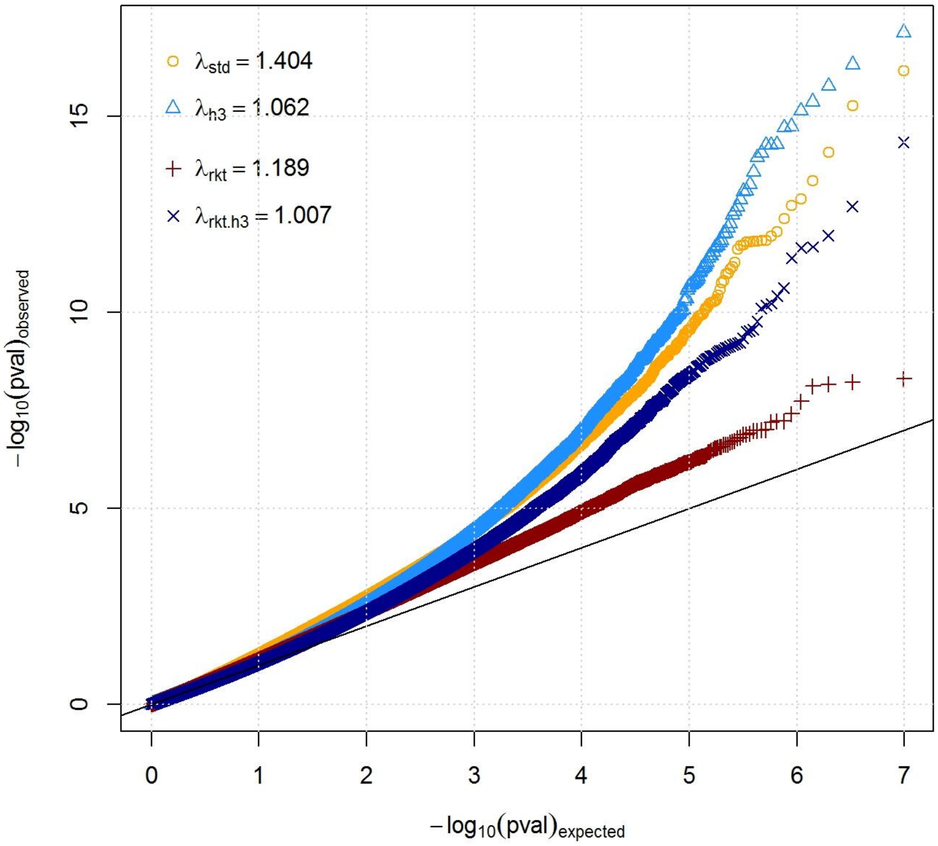

Supplement: S1 File — (PDF) [file pone.0173847.s001.pdf]
